# Supplementary material for: B. subtilis MutS2 splits stalled ribosomes into subunits without mRNA cleavage
Source: EMBO J. 2023 Dec 14;43(4):2. doi: 10.1038/s44318-023-00010-3 (PMC10897456; doi:10.1038/s44318-023-00010-3)
Supplement: Supplementary file 1 — Appendix Figure S1-S2 [file 44318_2023_10_MOESM1_ESM.pdf]

Appendix

B. subtilis MutS2 splits stalled ribosomes into subunits without mRNA cleavage

Esther Park1, Timur Mackens-Kiani2, Rebekah Berhane1, Hanna Esser2, Chimeg Erdenebat2, A. Maxwell Burroughs3, Otto Berninghausen2, L. Aravind3, Roland Beckmann2, Rachel Green1,4, Allen R. Buskirk1,*

Table of Contents:

Appendix Figure S1 and S2 page 1

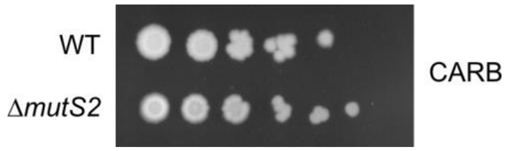

Appendix Figure S1. Spotting assay showing that *ΔmutS2* cells are not hypersensitive to carbenicillin (0.05 μg/mL).

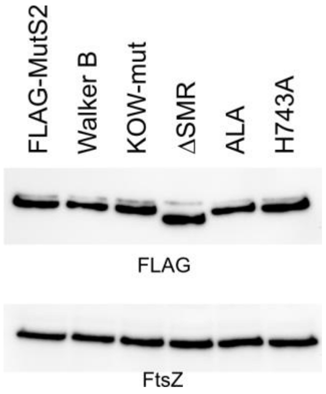

Appendix Figure S2. Expression levels of MutS2 mutants are similar. Levels of FLAG-tagged constructs of MutS2 in *B. subtilis* cells were detected on a western blot using an anti-FLAG antibody. The FtsZ protein serves as a loading control.
