# Supplementary figures and images for: B. subtilis MutS2 splits stalled ribosomes into subunits without mRNA cleavage
Source: EMBO J. 2023 Dec 14;43(4):2. doi: 10.1038/s44318-023-00010-3 (PMC10897456; doi:10.1038/s44318-023-00010-3)

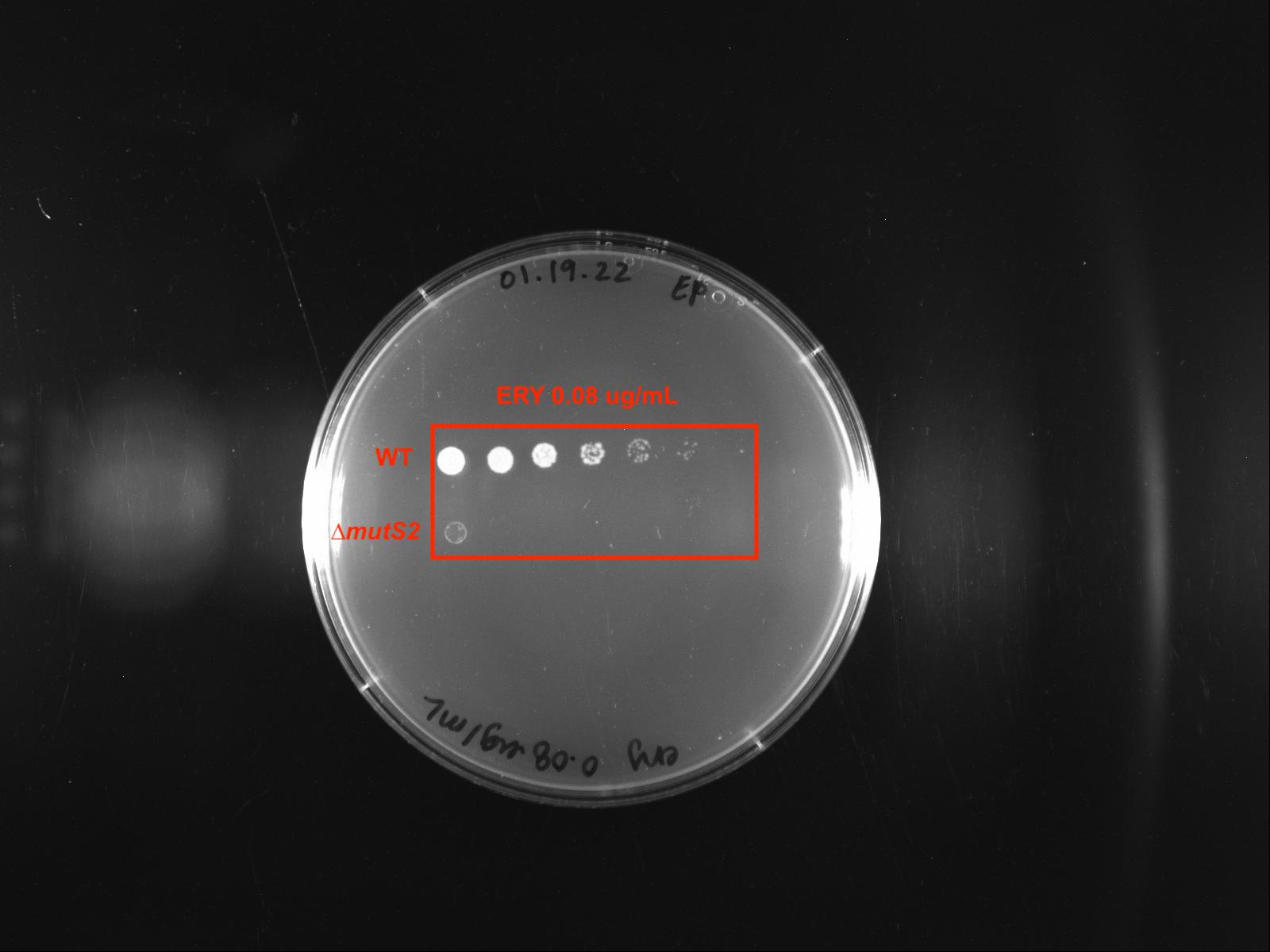

Supplement: Supplementary file 3 — Source Data Fig. 1 [file 44318_2023_10_MOESM3_ESM.zip › Figure 1/1C/1C_Spotting_ERY.Jpeg]

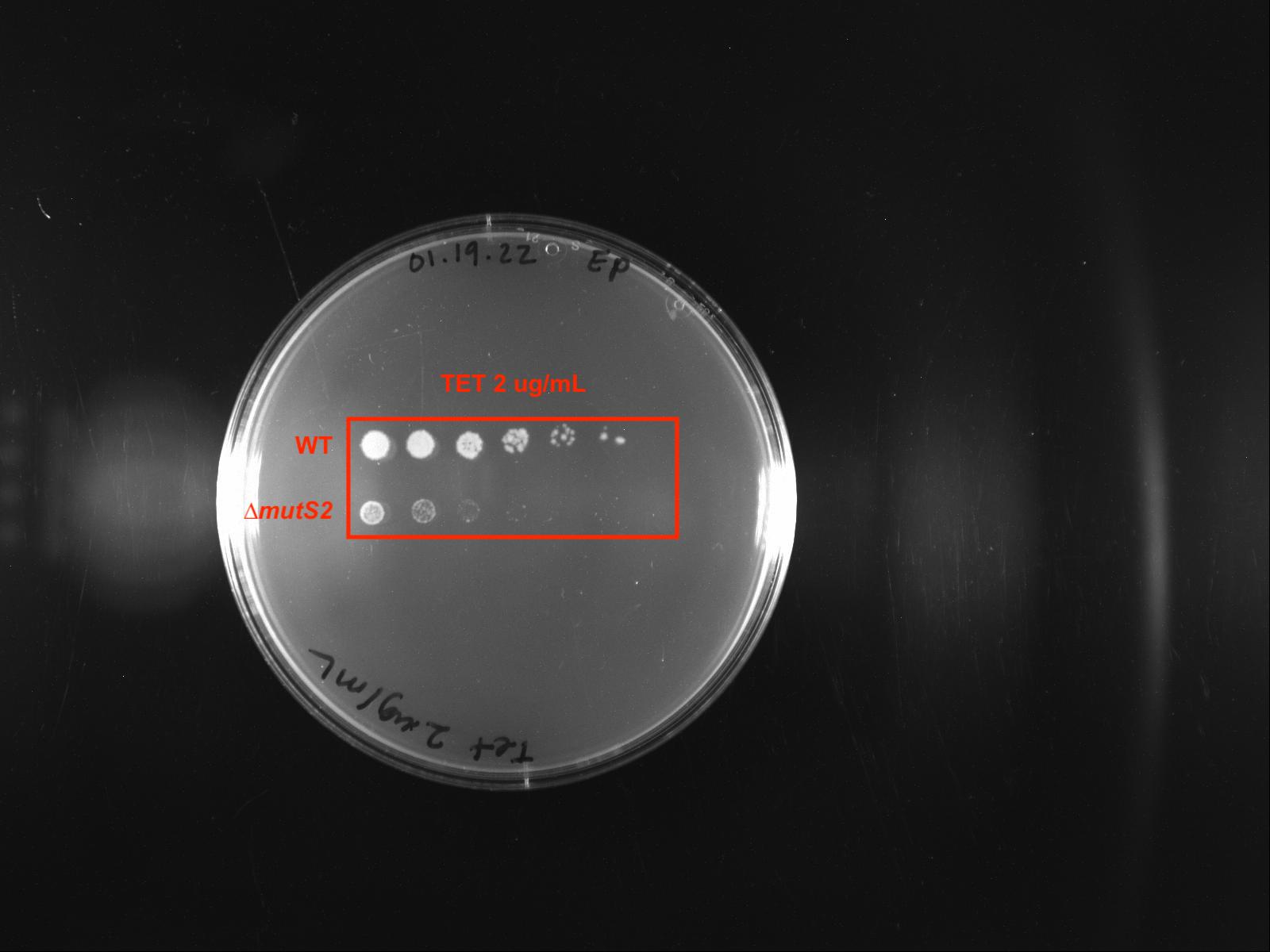

Supplement: Supplementary file 3 — Source Data Fig. 1 [file 44318_2023_10_MOESM3_ESM.zip › Figure 1/1C/1C_Spotting_TET.Jpeg]

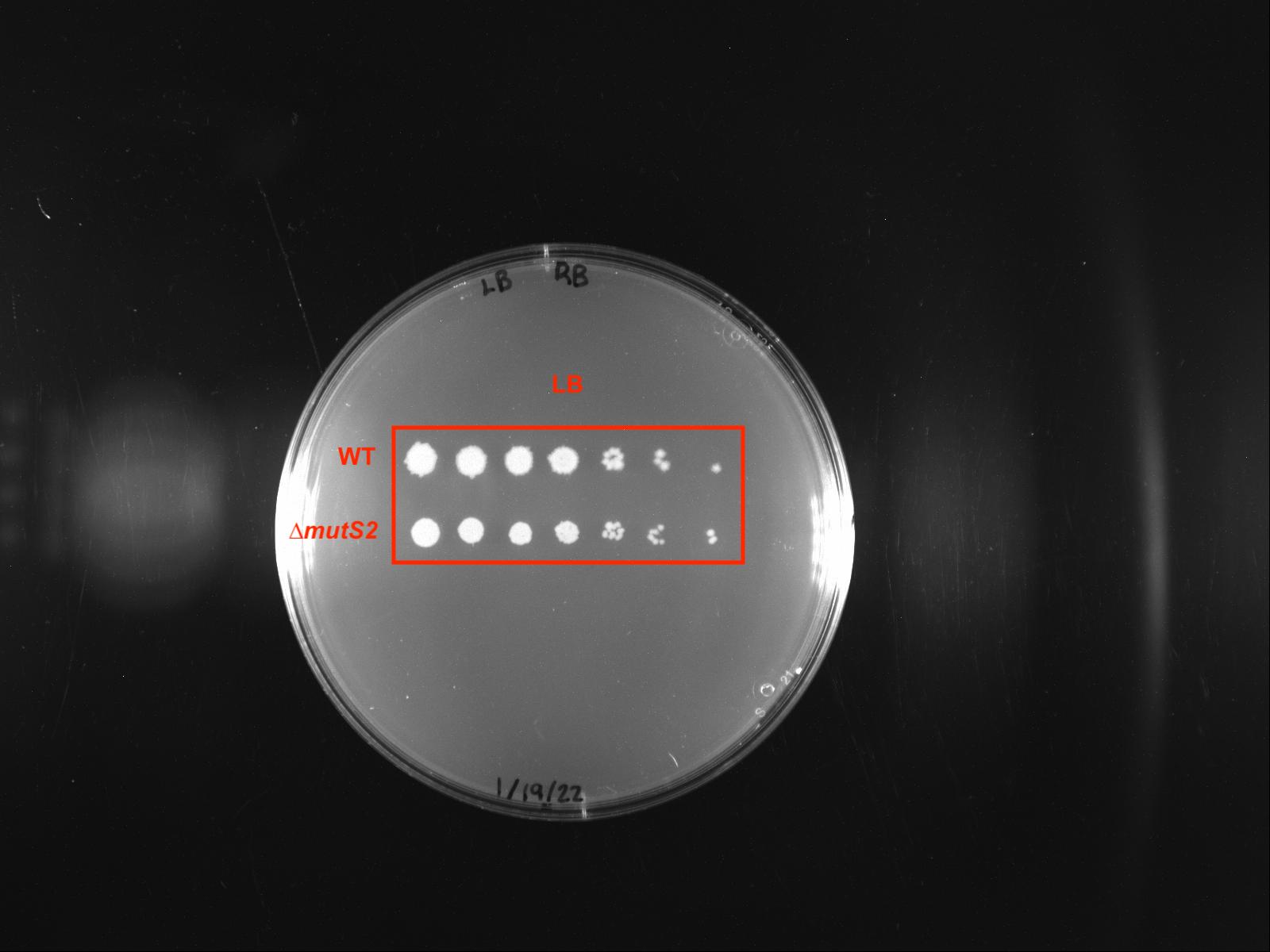

Supplement: Supplementary file 3 — Source Data Fig. 1 [file 44318_2023_10_MOESM3_ESM.zip › Figure 1/1C/1C_Spotting_LB.Jpeg]

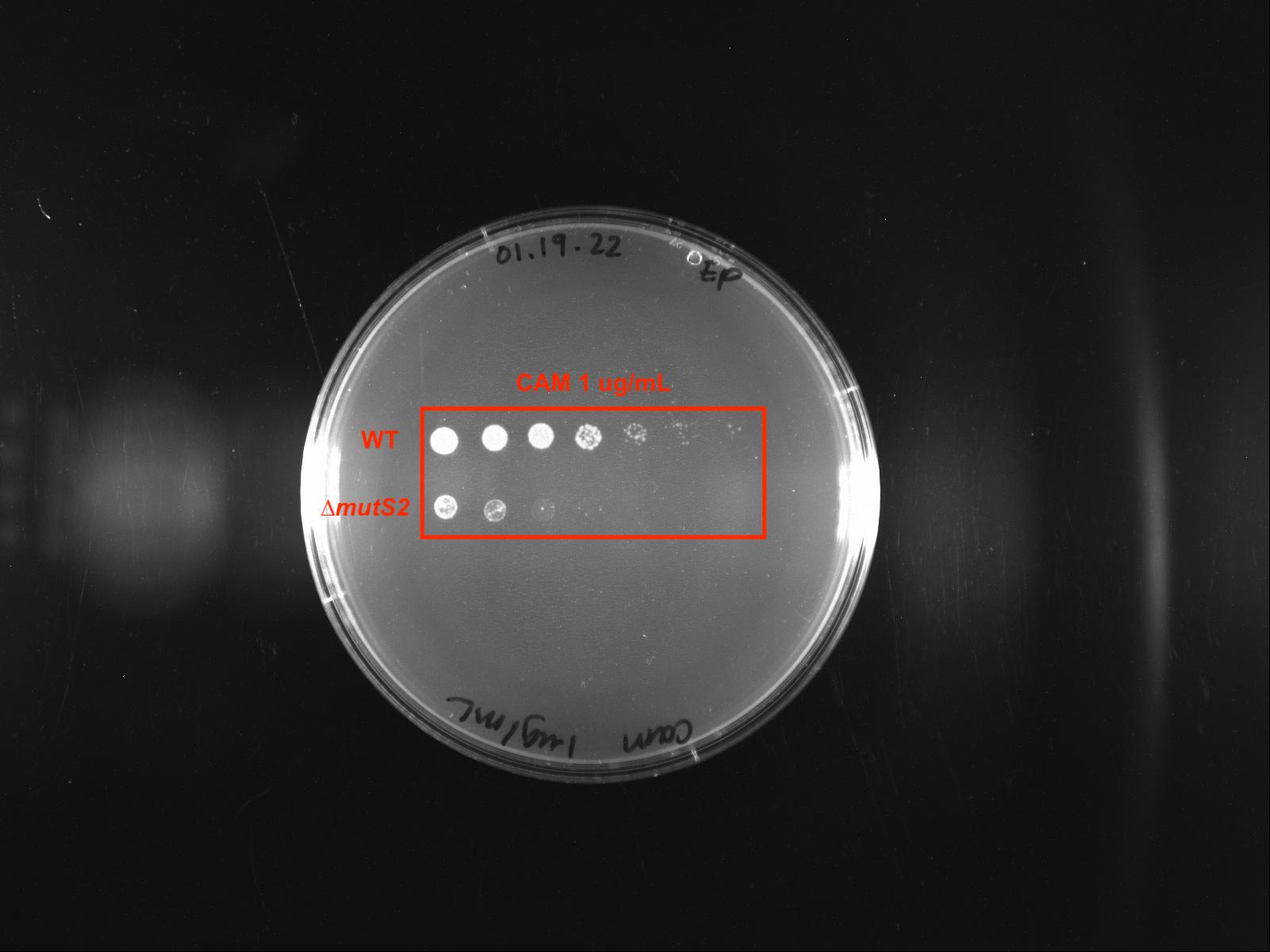

Supplement: Supplementary file 3 — Source Data Fig. 1 [file 44318_2023_10_MOESM3_ESM.zip › Figure 1/1C/1C_Spotting_CAM.Jpeg]

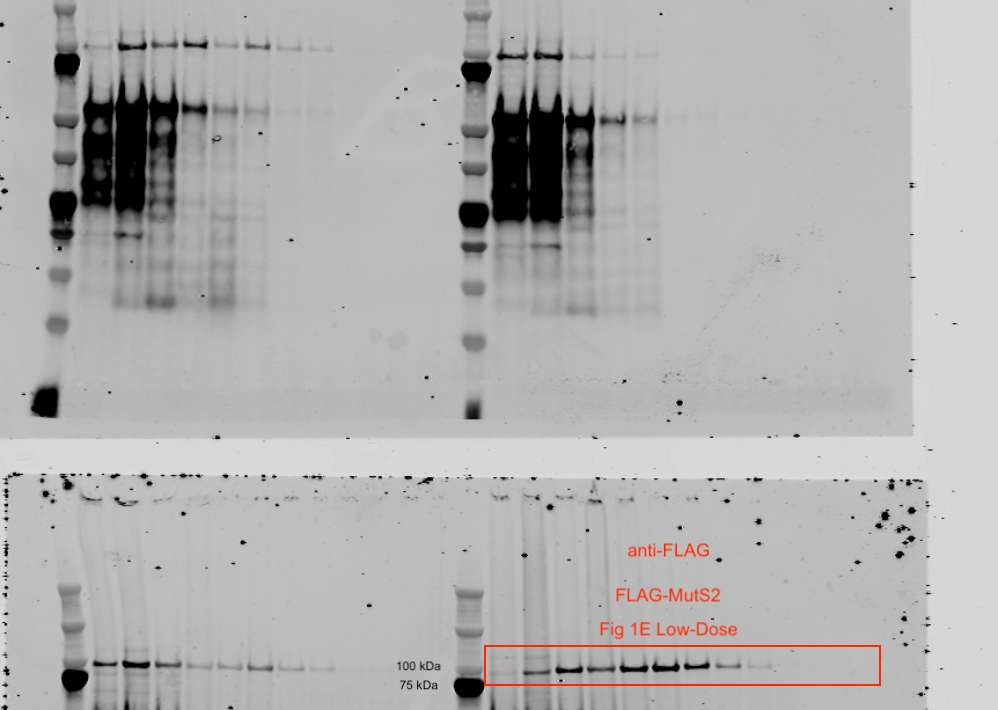

Supplement: Supplementary file 3 — Source Data Fig. 1 [file 44318_2023_10_MOESM3_ESM.zip › Figure 1/1E/blots/Low_Dose_CAM.tif]

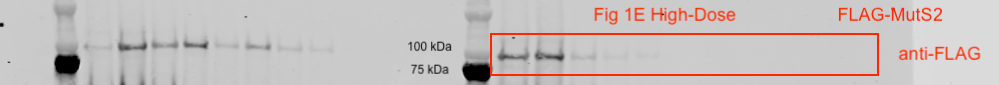

Supplement: Supplementary file 3 — Source Data Fig. 1 [file 44318_2023_10_MOESM3_ESM.zip › Figure 1/1E/blots/High_Dose_CAM.png]

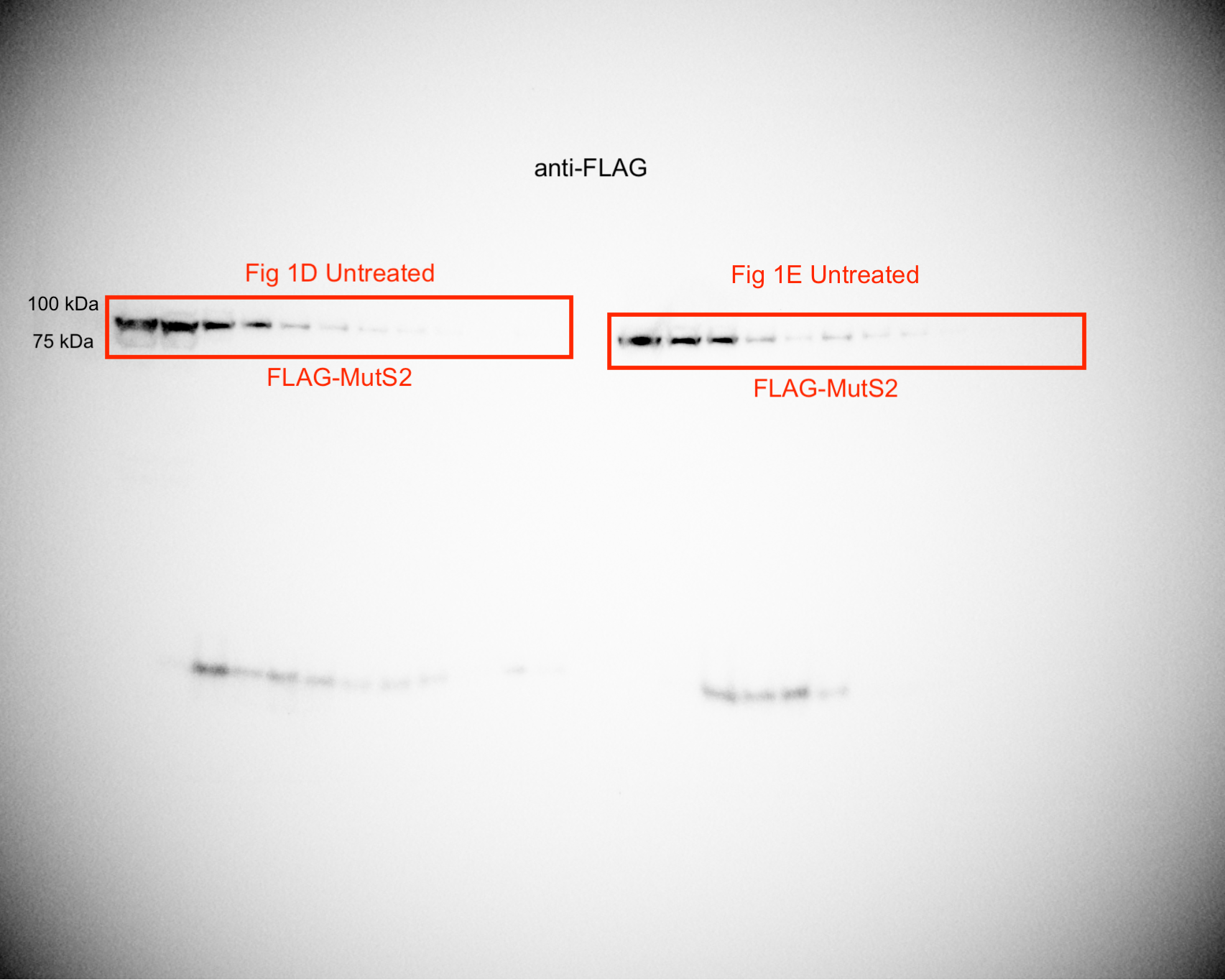

Supplement: Supplementary file 3 — Source Data Fig. 1 [file 44318_2023_10_MOESM3_ESM.zip › Figure 1/1E/blots/Untreated.tif]

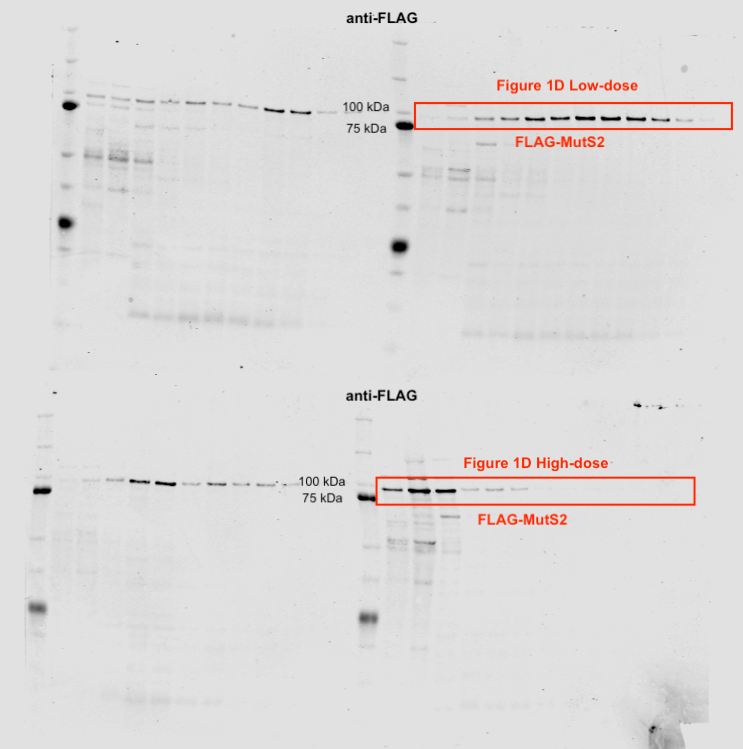

Supplement: Supplementary file 3 — Source Data Fig. 1 [file 44318_2023_10_MOESM3_ESM.zip › Figure 1/1D/blots/CAM.png]

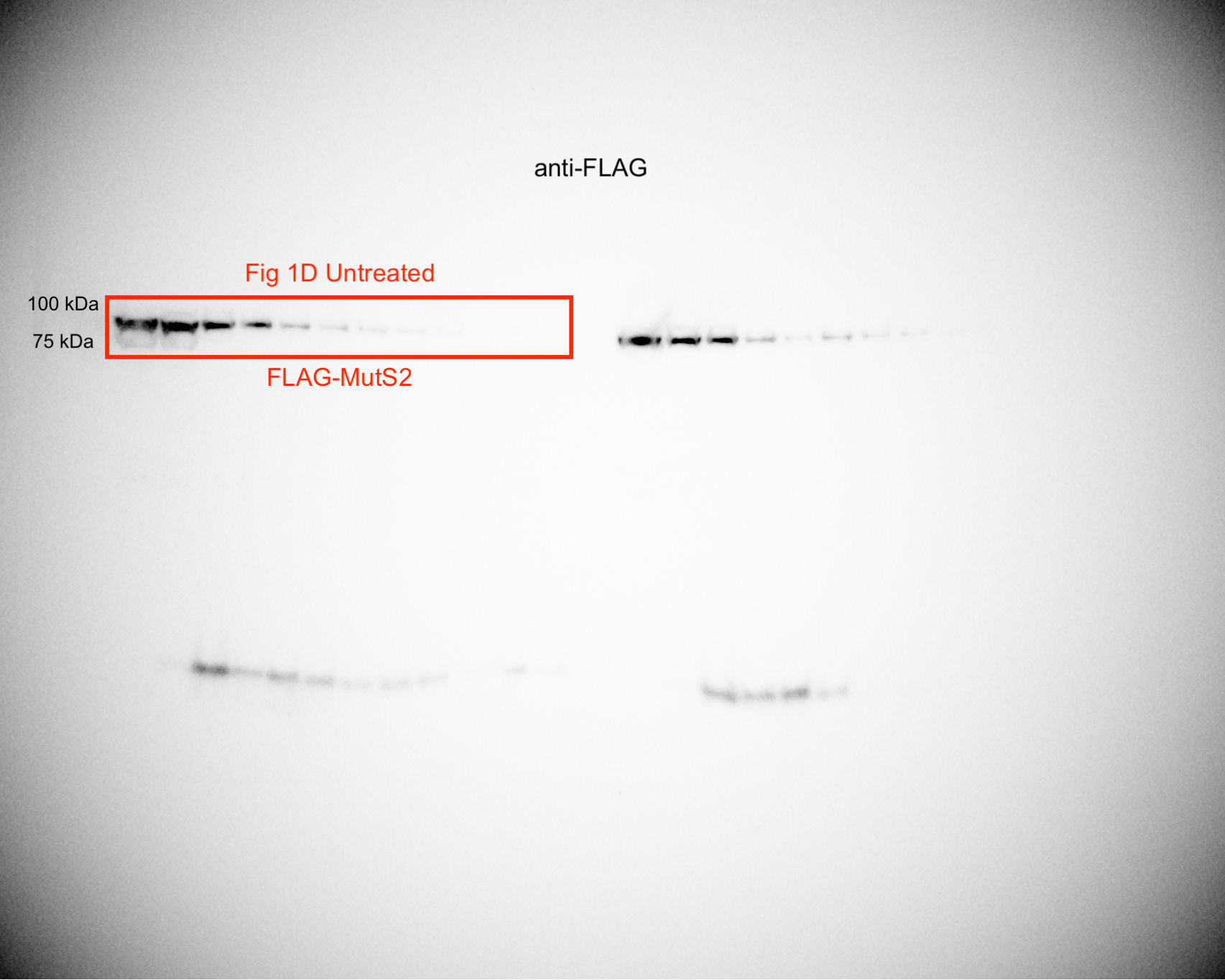

Supplement: Supplementary file 3 — Source Data Fig. 1 [file 44318_2023_10_MOESM3_ESM.zip › Figure 1/1D/blots/Untreated.tif]

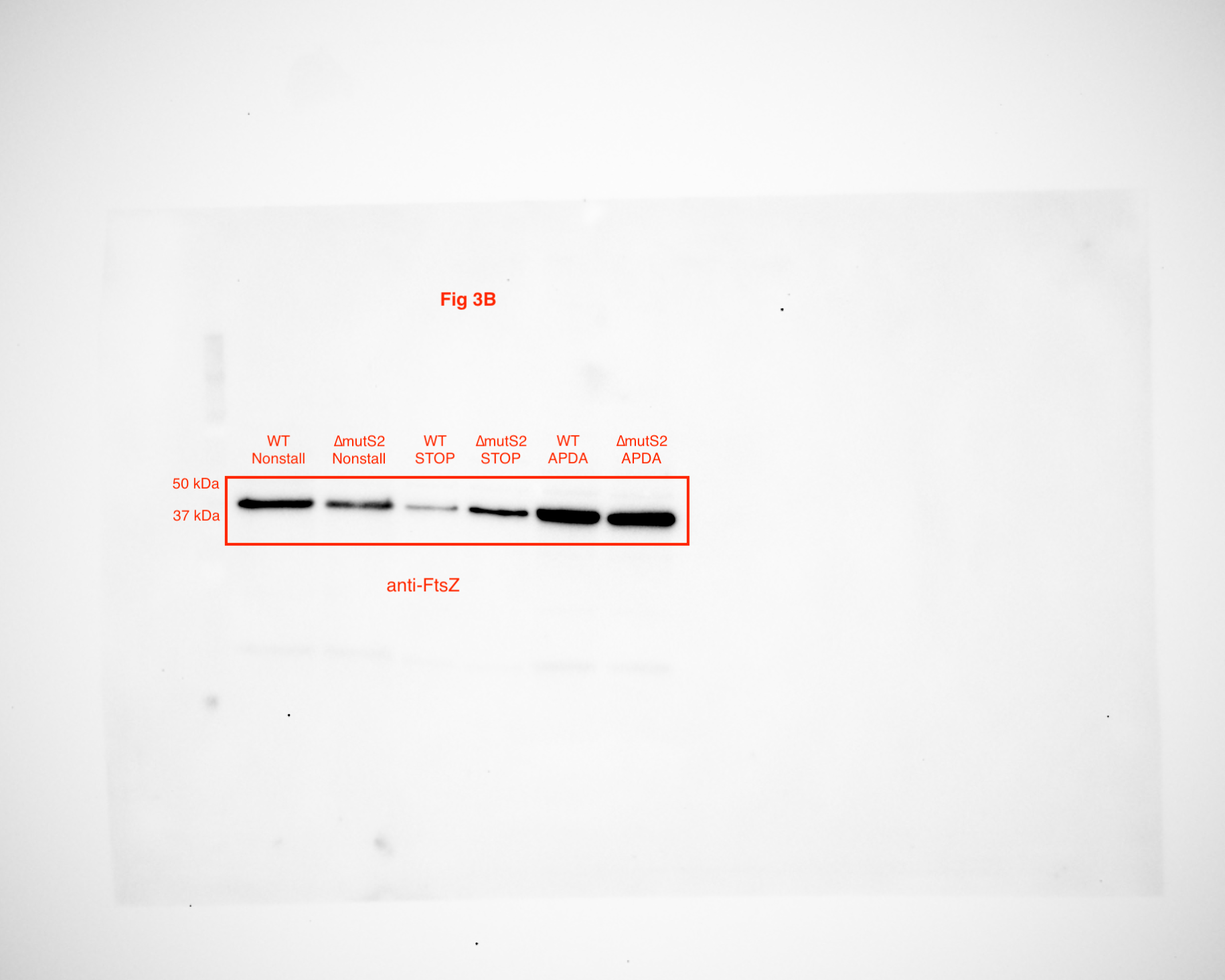

Supplement: Supplementary file 4 — Source Data Fig. 3 [file 44318_2023_10_MOESM4_ESM.zip › Figure 3/3B/3B_FtsZ.tif]

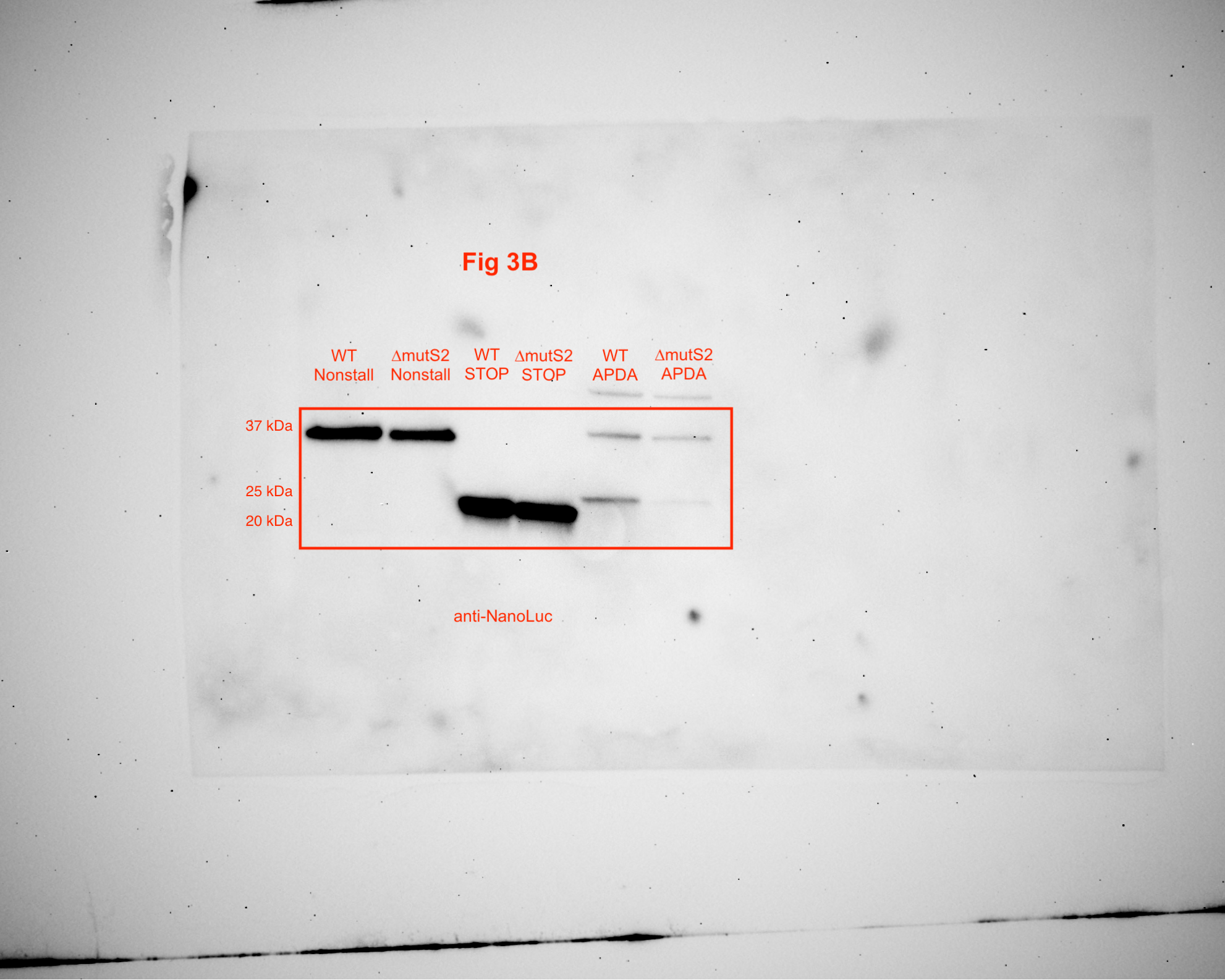

Supplement: Supplementary file 4 — Source Data Fig. 3 [file 44318_2023_10_MOESM4_ESM.zip › Figure 3/3B/3B_Reporter_Western.tif]

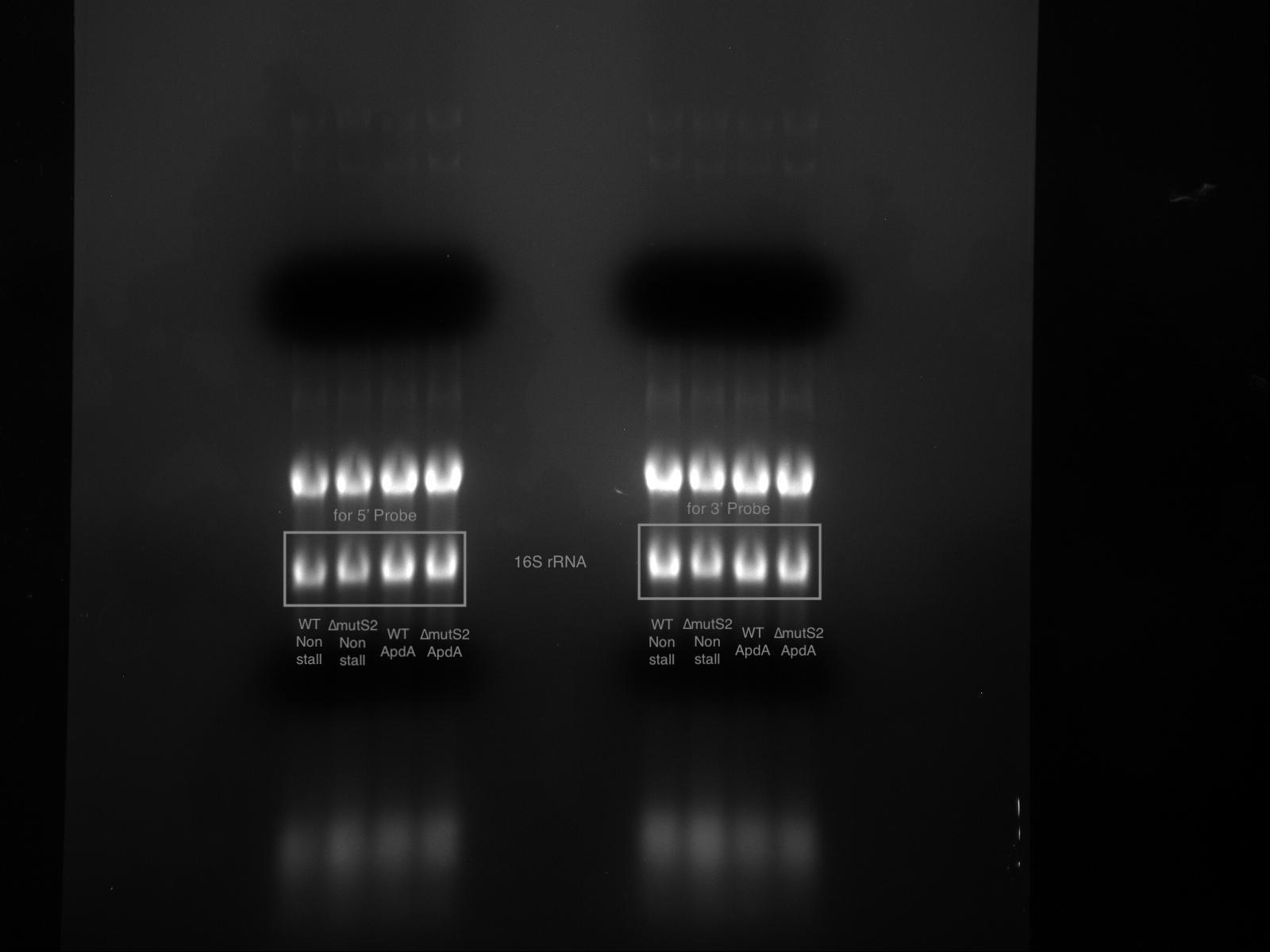

Supplement: Supplementary file 4 — Source Data Fig. 3 [file 44318_2023_10_MOESM4_ESM.zip › Figure 3/3C/16S rRNA.Jpeg]

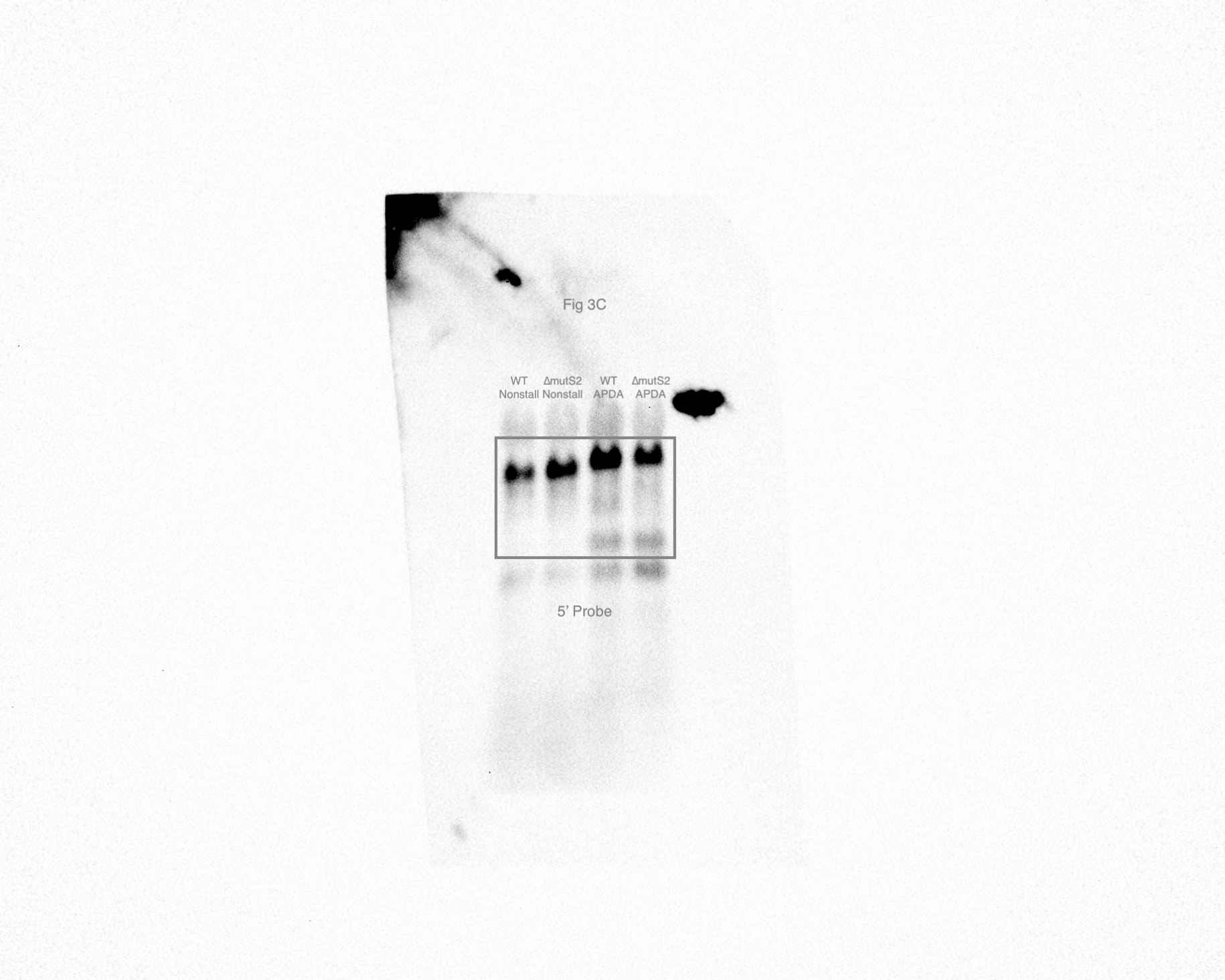

Supplement: Supplementary file 4 — Source Data Fig. 3 [file 44318_2023_10_MOESM4_ESM.zip › Figure 3/3C/3C_5'.tif]

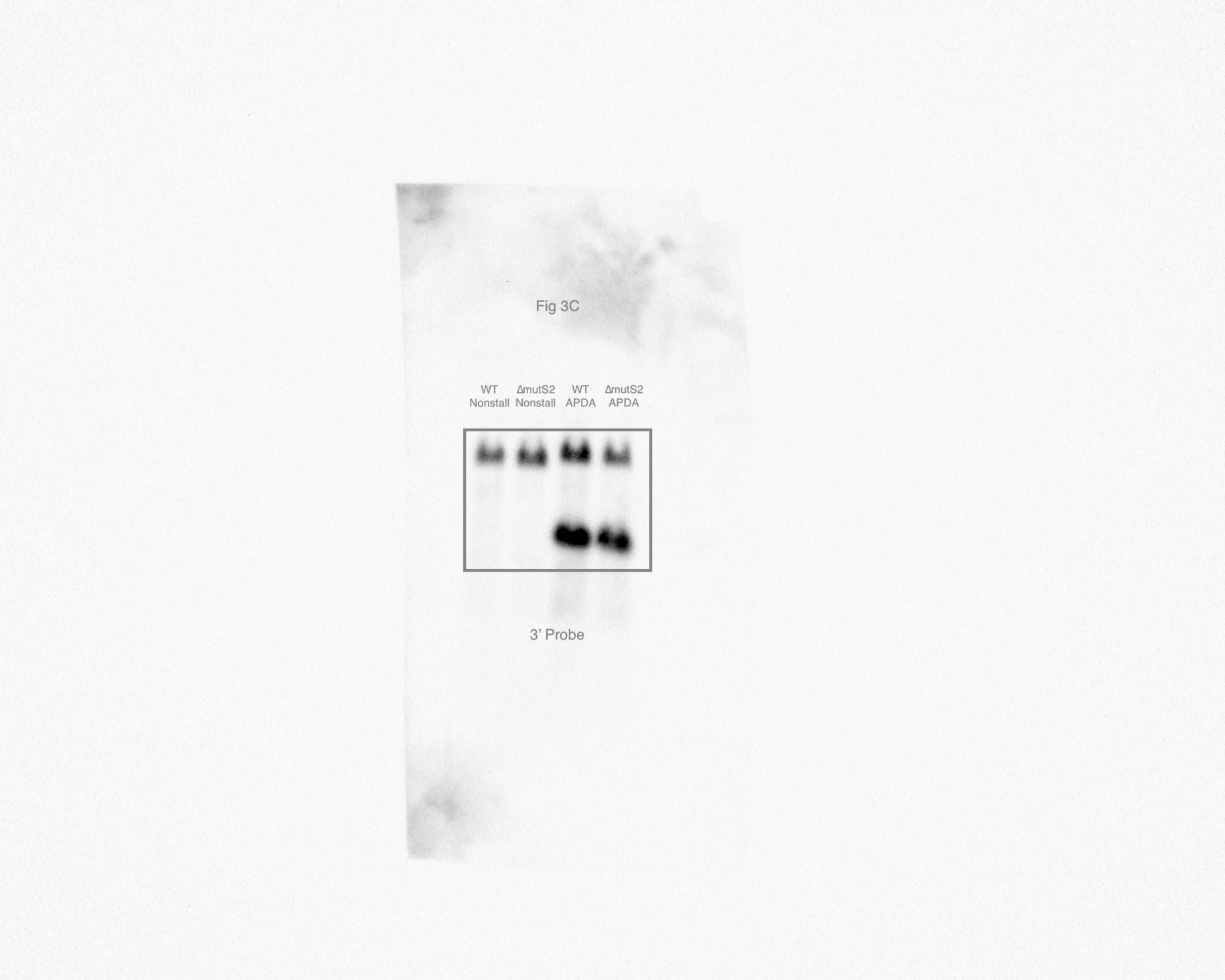

Supplement: Supplementary file 4 — Source Data Fig. 3 [file 44318_2023_10_MOESM4_ESM.zip › Figure 3/3C/3C_3'.tif]

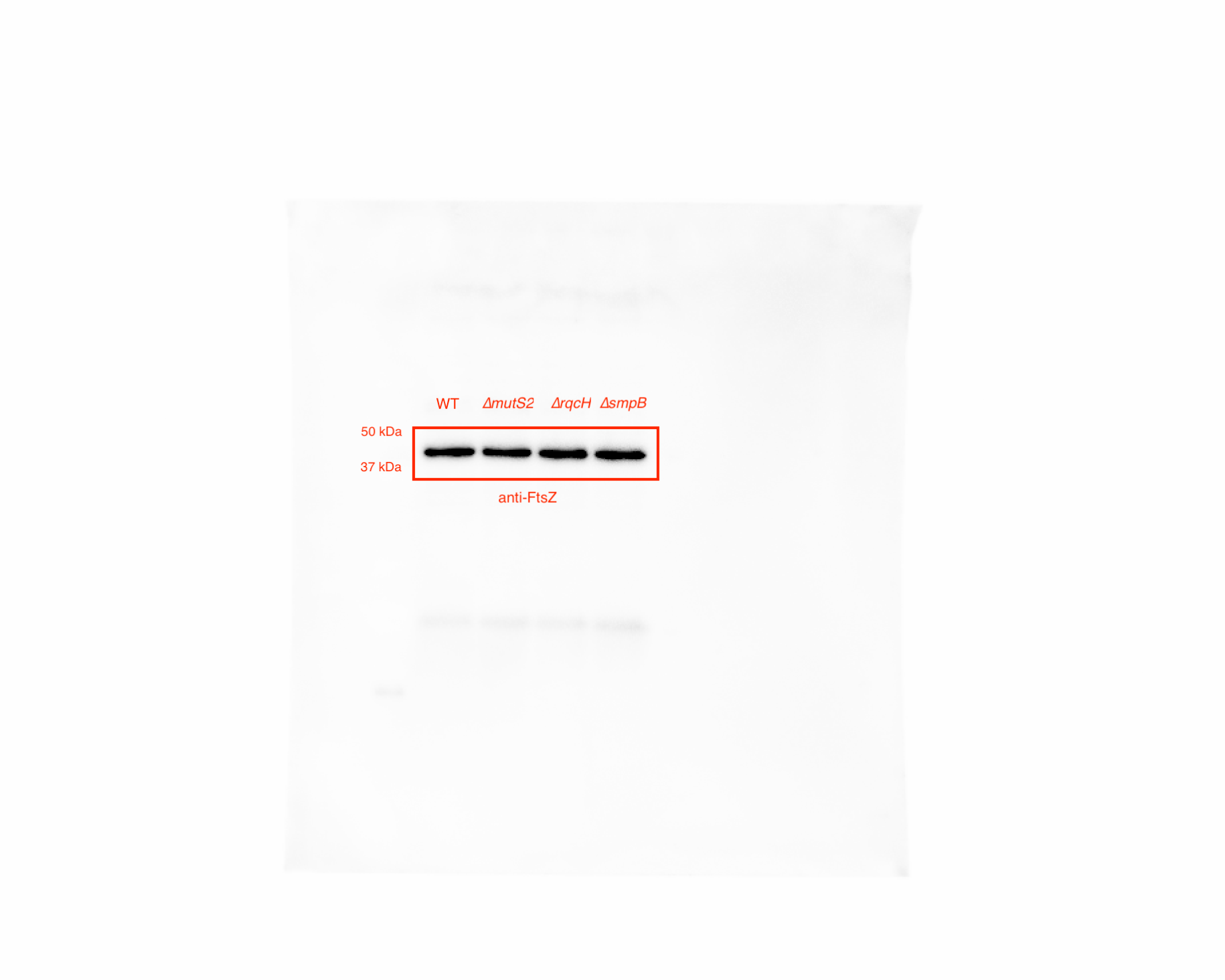

Supplement: Supplementary file 4 — Source Data Fig. 3 [file 44318_2023_10_MOESM4_ESM.zip › Figure 3/3D/FtsZ.tif]

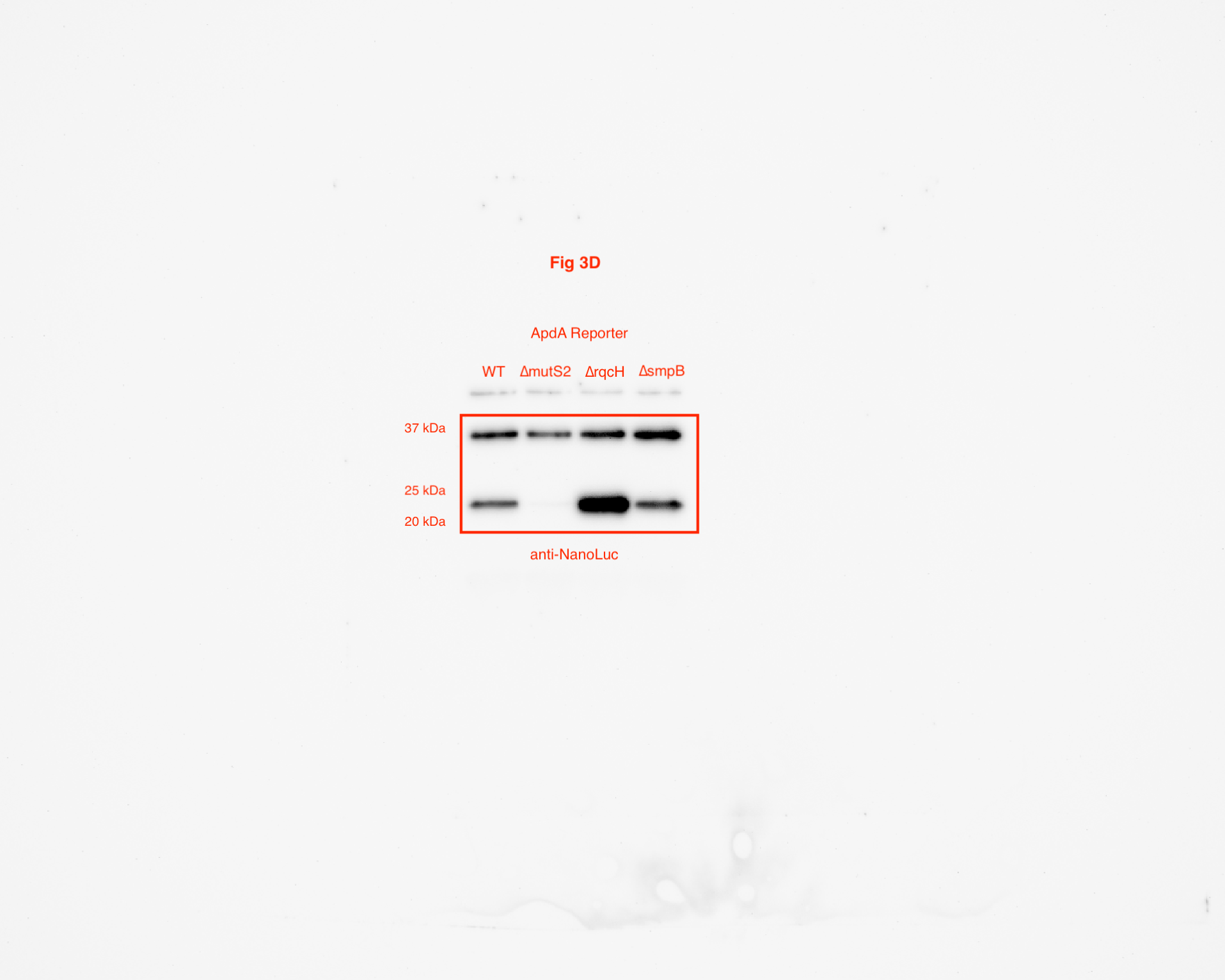

Supplement: Supplementary file 4 — Source Data Fig. 3 [file 44318_2023_10_MOESM4_ESM.zip › Figure 3/3D/Fig3D_western.tif]

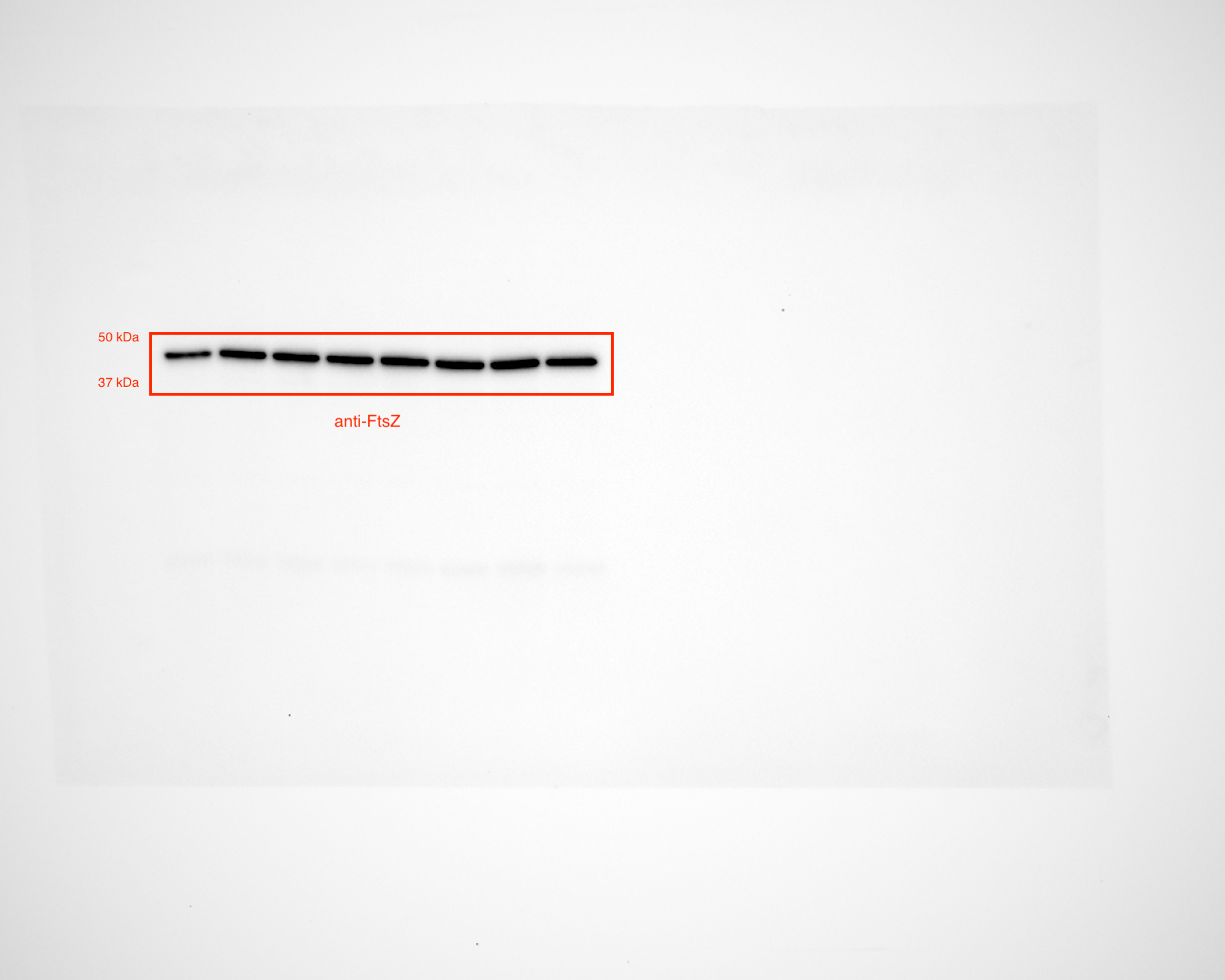

Supplement: Supplementary file 5 — Source Data Fig. 4 [file 44318_2023_10_MOESM5_ESM.zip › Figure 4/4B/FtsZ.tif]

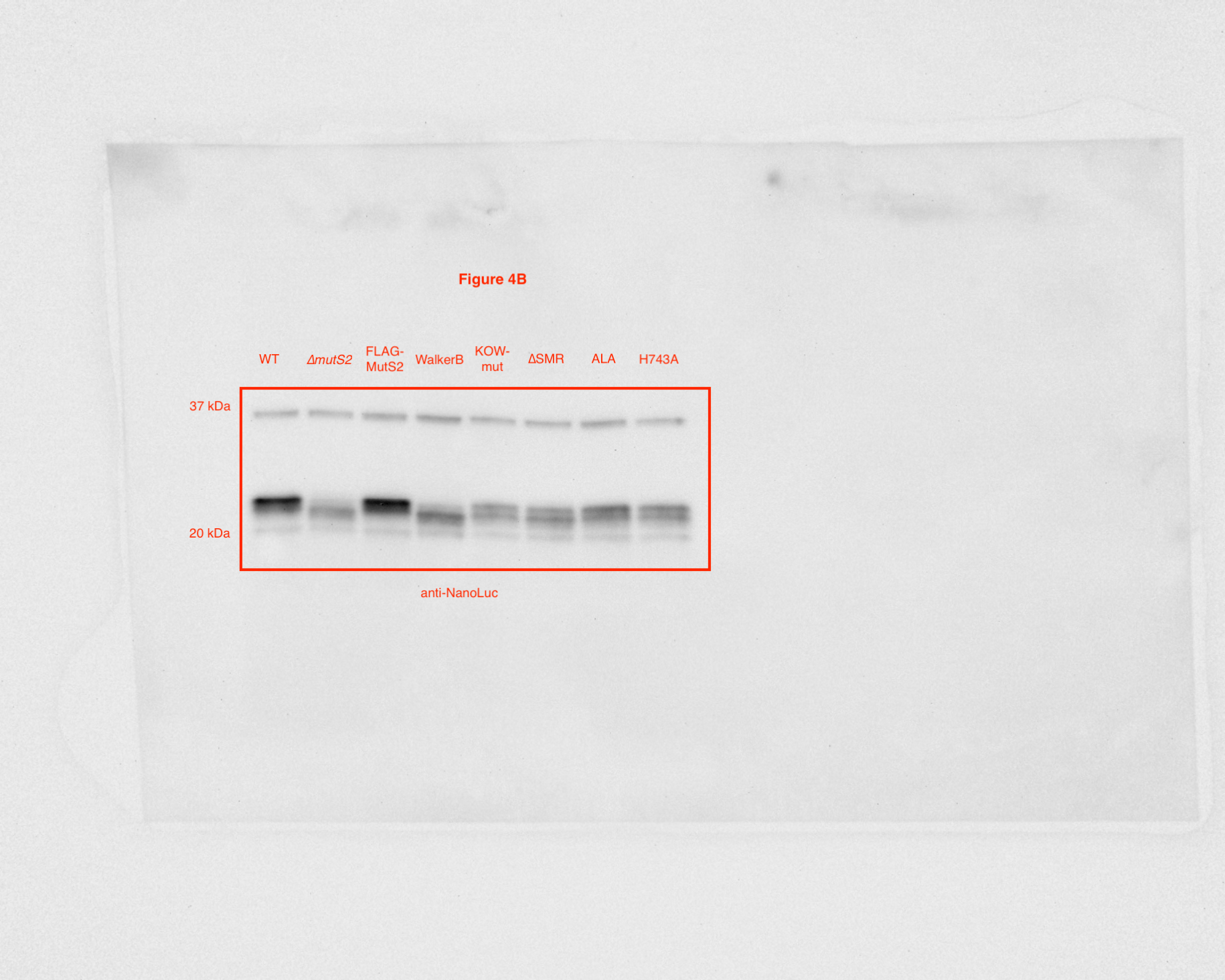

Supplement: Supplementary file 5 — Source Data Fig. 4 [file 44318_2023_10_MOESM5_ESM.zip › Figure 4/4B/Reporter Western.tif]

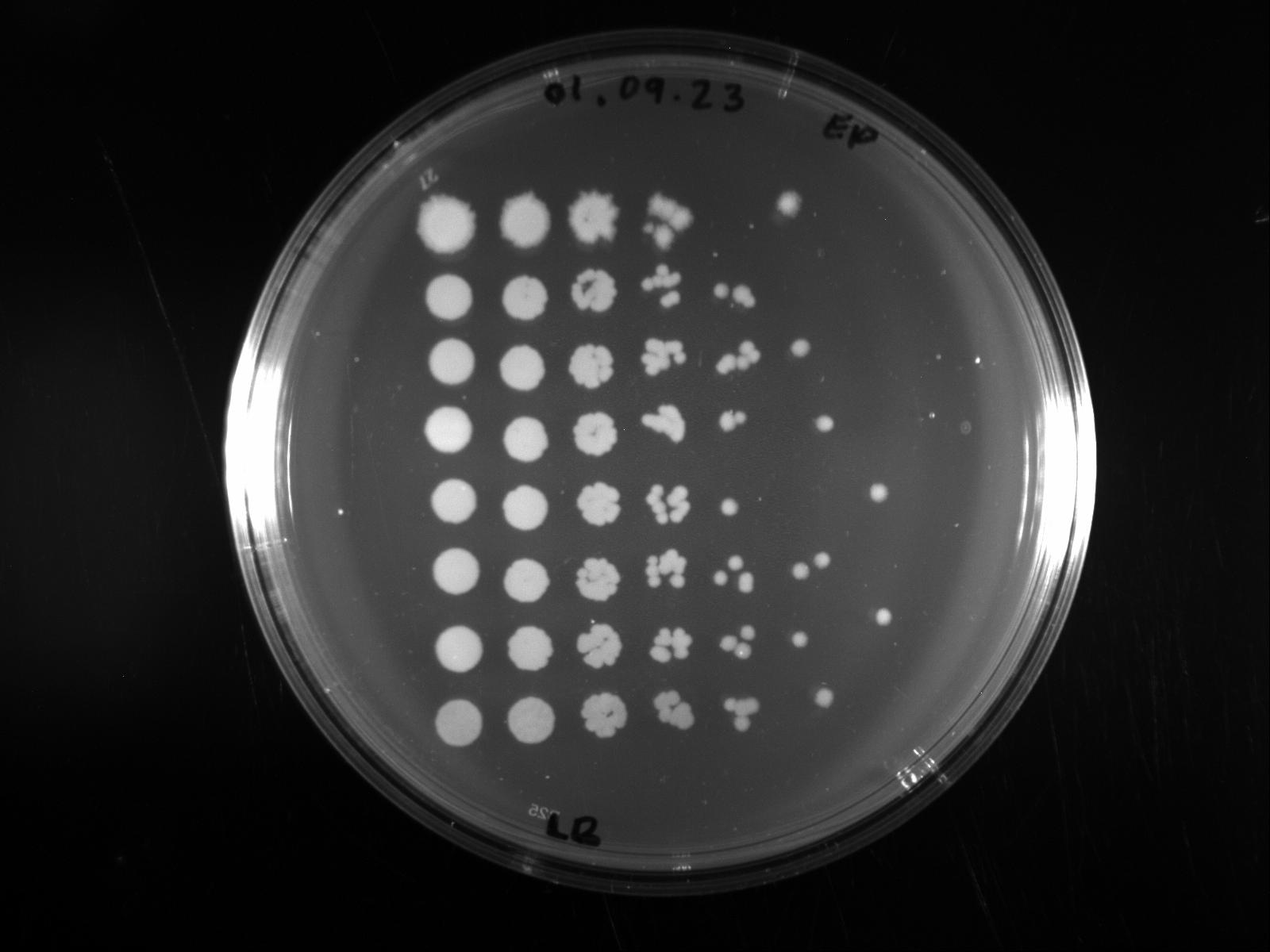

Supplement: Supplementary file 5 — Source Data Fig. 4 [file 44318_2023_10_MOESM5_ESM.zip › Figure 4/4C/LB.Jpeg]

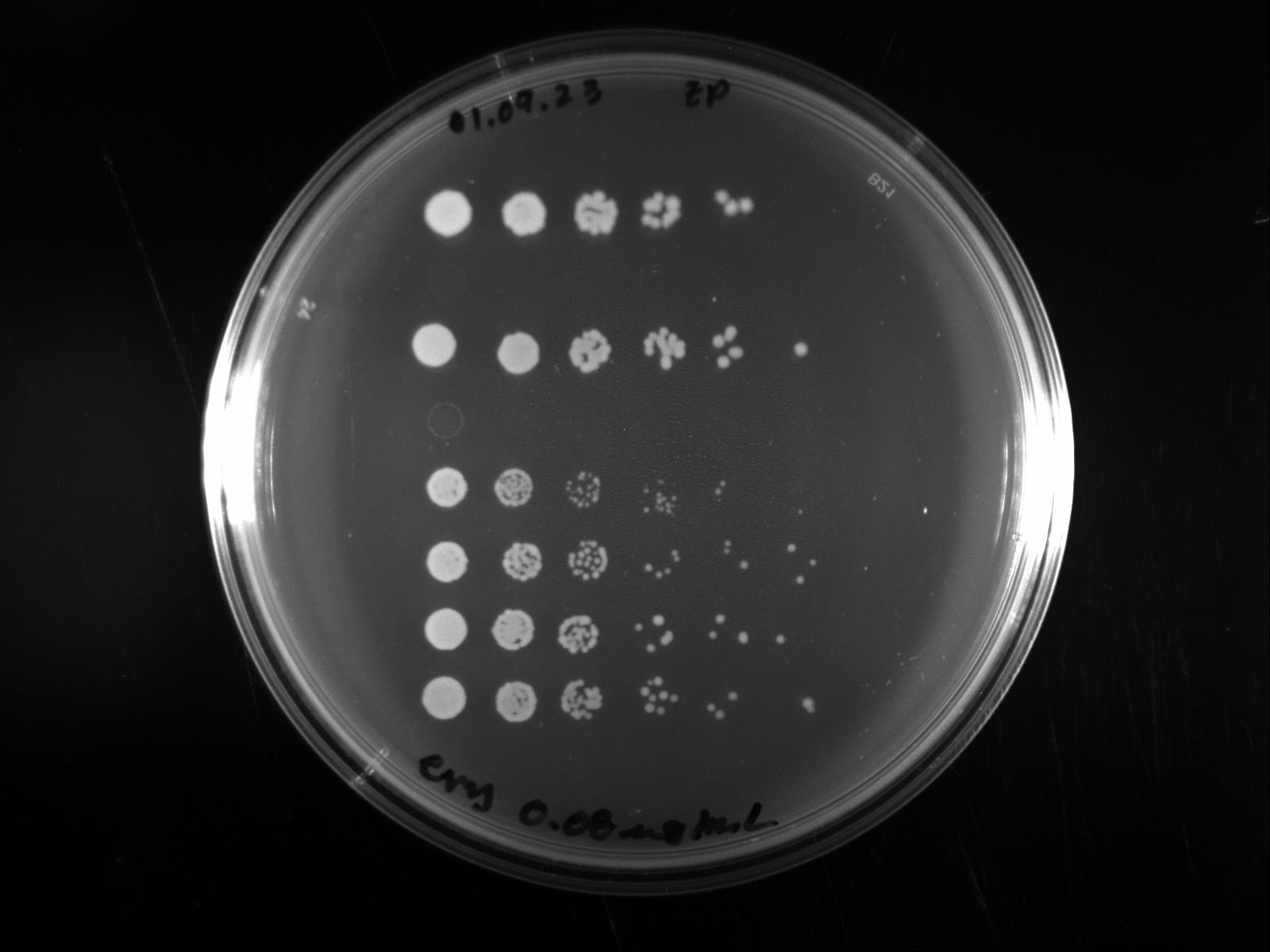

Supplement: Supplementary file 5 — Source Data Fig. 4 [file 44318_2023_10_MOESM5_ESM.zip › Figure 4/4C/ERY.Jpeg]

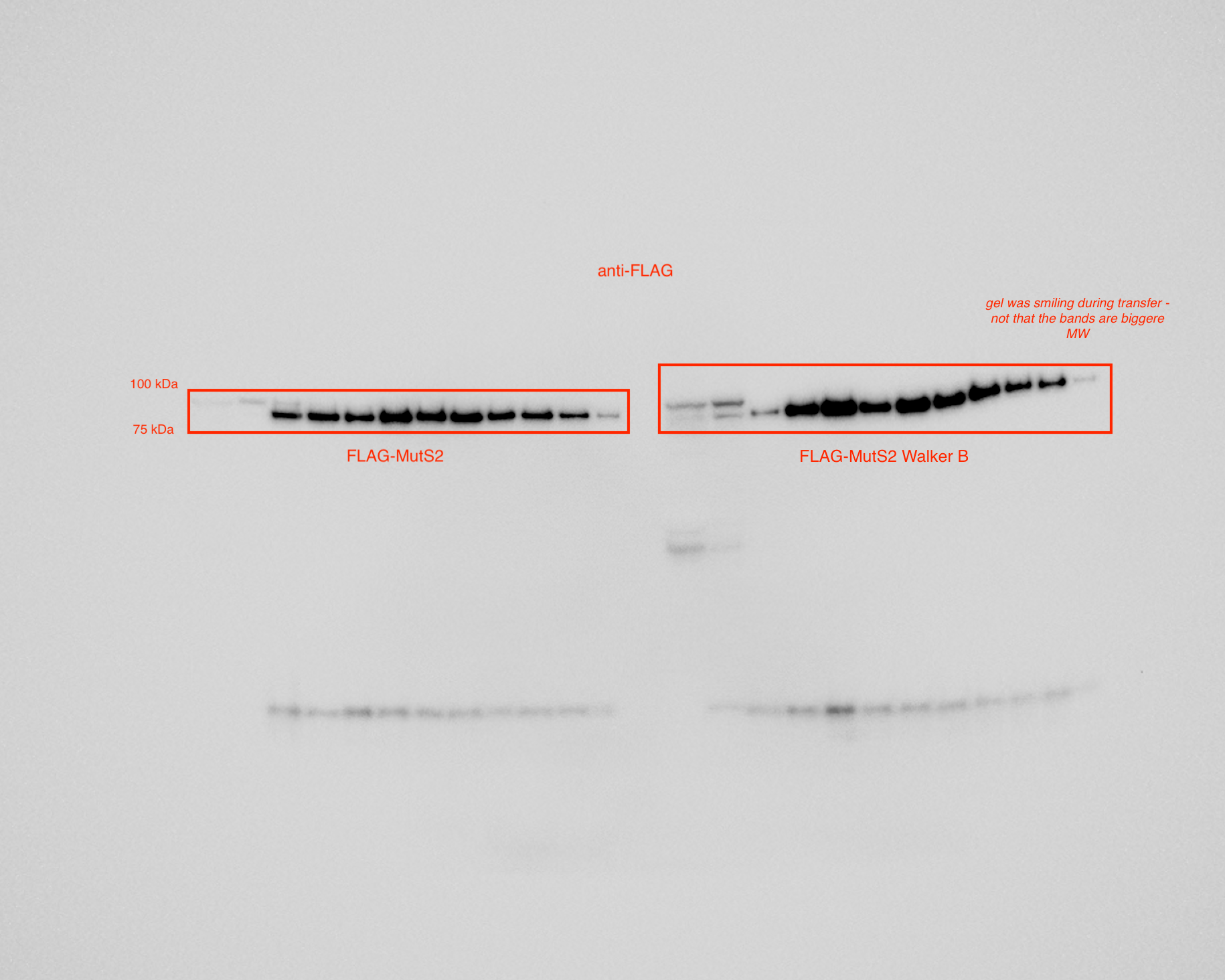

Supplement: Supplementary file 5 — Source Data Fig. 4 [file 44318_2023_10_MOESM5_ESM.zip › Figure 4/4A/blots/WTandWalkerB_gradient_western.tif]

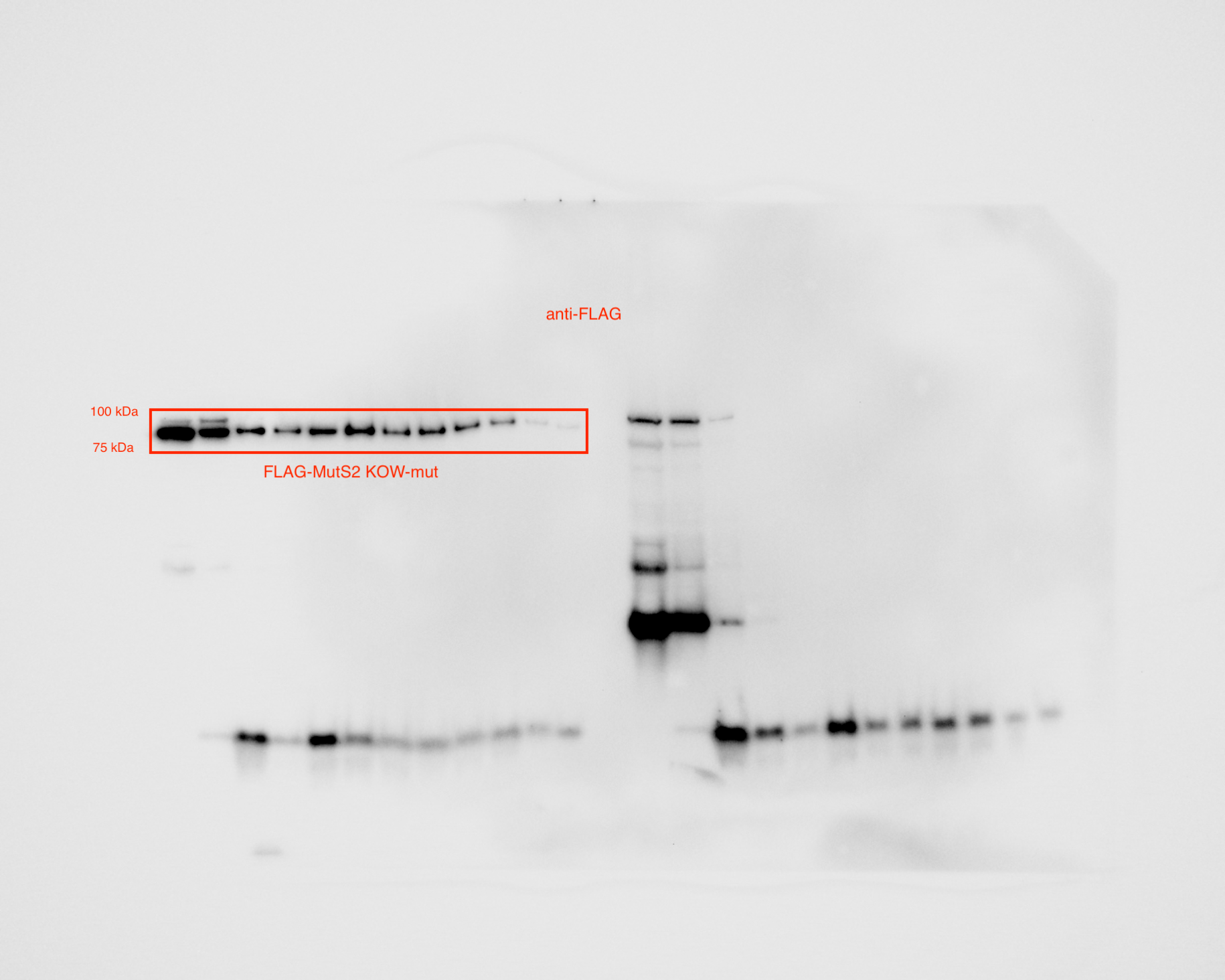

Supplement: Supplementary file 5 — Source Data Fig. 4 [file 44318_2023_10_MOESM5_ESM.zip › Figure 4/4A/blots/KOW-mut_gradient_western.tif]

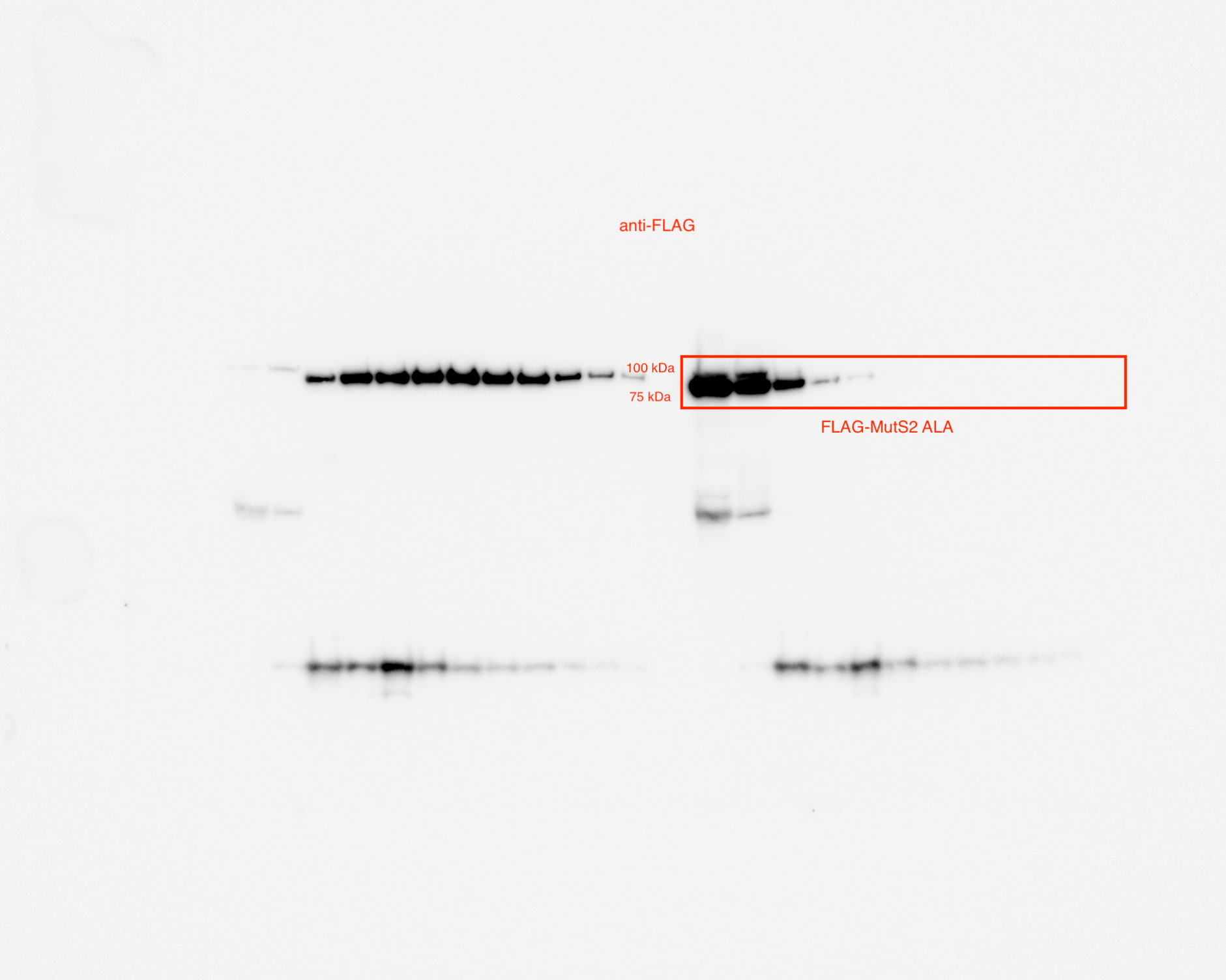

Supplement: Supplementary file 5 — Source Data Fig. 4 [file 44318_2023_10_MOESM5_ESM.zip › Figure 4/4A/blots/ALA_gradient_western.tif]

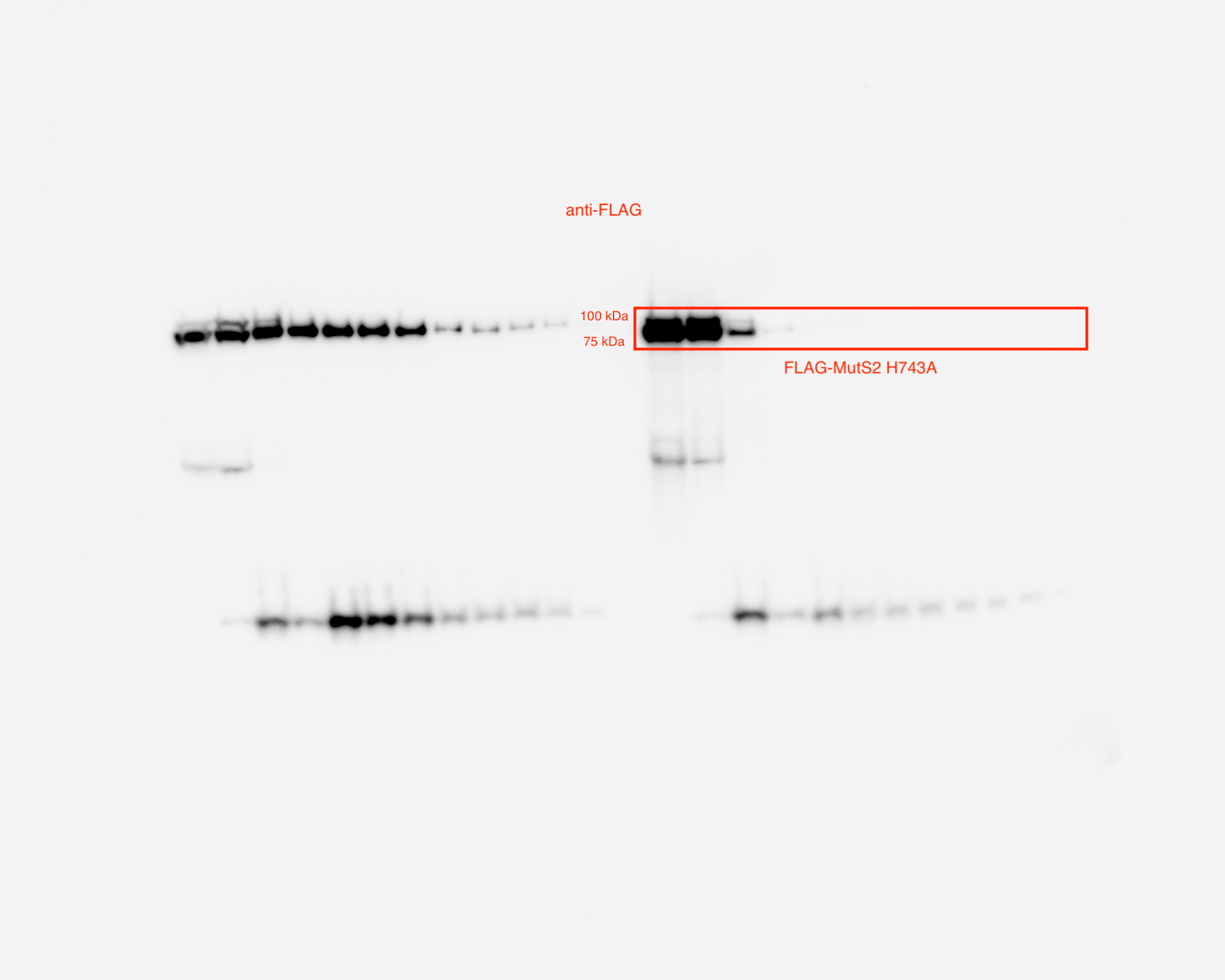

Supplement: Supplementary file 5 — Source Data Fig. 4 [file 44318_2023_10_MOESM5_ESM.zip › Figure 4/4A/blots/H743A_gradient_western.tif]

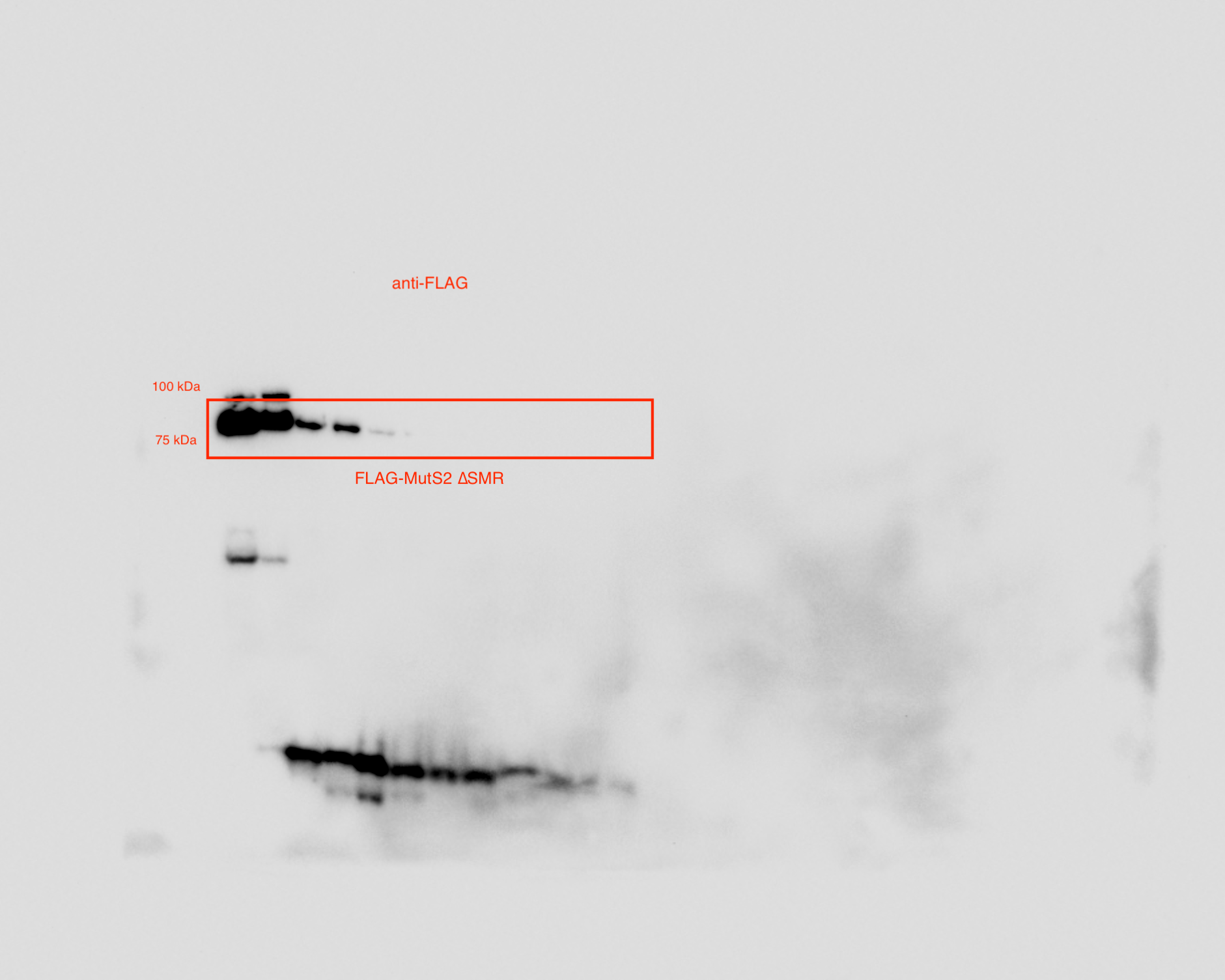

Supplement: Supplementary file 5 — Source Data Fig. 4 [file 44318_2023_10_MOESM5_ESM.zip › Figure 4/4A/blots/noSMR_gradient_western.tif]
