# Supplementary material for: B. subtilis MutS2 splits stalled ribosomes into subunits without mRNA cleavage
Source: EMBO J. 2023 Dec 14;43(4):2. doi: 10.1038/s44318-023-00010-3 (PMC10897456; doi:10.1038/s44318-023-00010-3)
Supplement: Supplementary file 7 — Expanded View Figures [file 44318_2023_10_MOESM7_ESM.pdf]

Expanded View Figures

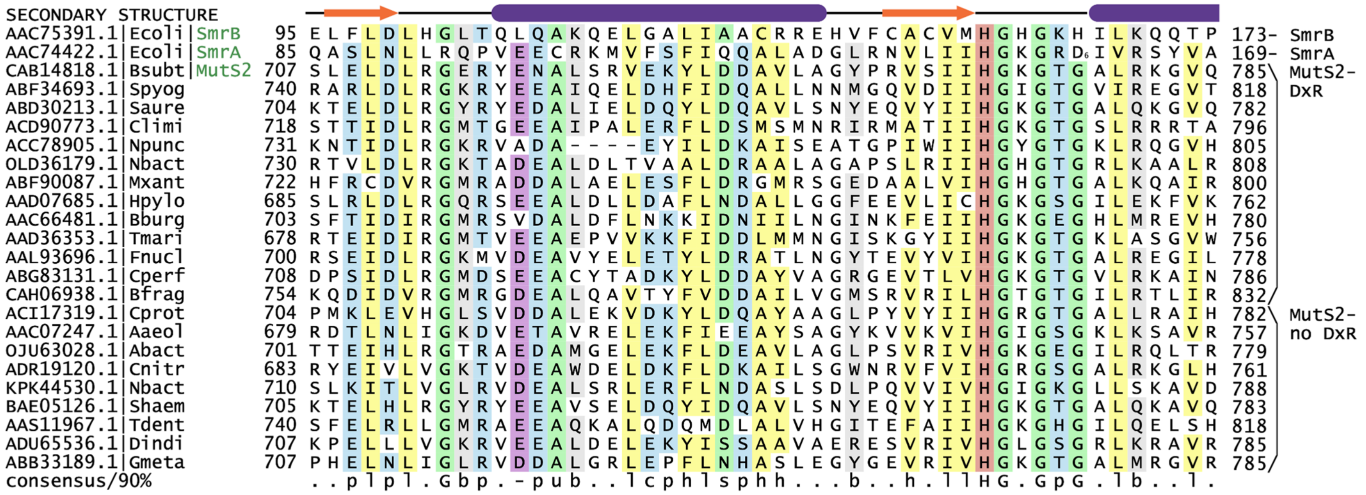

Figure EV1. Multiple alignment of the conserved residues in the SMR domains from different bacteria.

Columns in the alignment are shaded and labeled according to biochemical character: −, negatively charged in purple; c, charged in blue; h, hydrophobic in yellow; p, polar in blue; l, aliphatic in yellow; b, big in gray; s, small in green; u, tiny in green; G, glycine in green; H, histidine in red. Sequences are labeled with NCBI accession number and organism abbreviation. Secondary structure provided at top of alignment. Numbers to left and right of alignment denote positioning of the region.

© The Author(s)

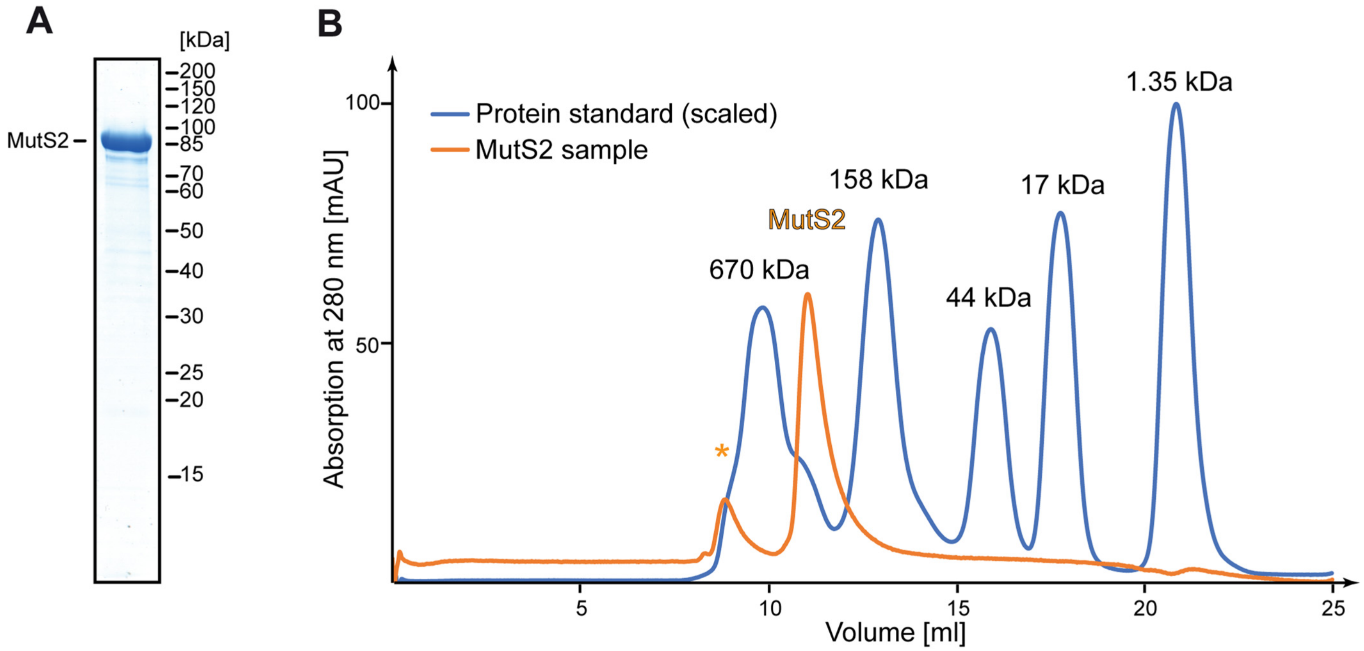

Figure EV2. MutS2 purification as an oligomer.

(**A**) Purified sample of *B. subtilis* MutS2 used for reconstitution experiments, shown on a Coomassie-stained 10% Nu-PAGE gel. The expected apparent molecular weight of MutS2 is approximately 87 kDa. (**B**) Chromatograms of size-exclusion chromatography with the purified MutS2 sample (orange) and a protein size standard (blue) consisting of Thyroglobulin (bovine, 670 kDA), g-globulin (bovine, 158 kDa), Ovalbumin (chicken, 44 kDa), Myoglobin (horse, 17 kDa), and Vitamin B12 (1,35 kDa). Samples were analyzed on a Superdex 200 increase 10/300 GL column. Results indicate a single purified MutS2 complex of a size between 158 kDa and 670 kDa, consistent with a dimer or tetramer.

© The Author(s)

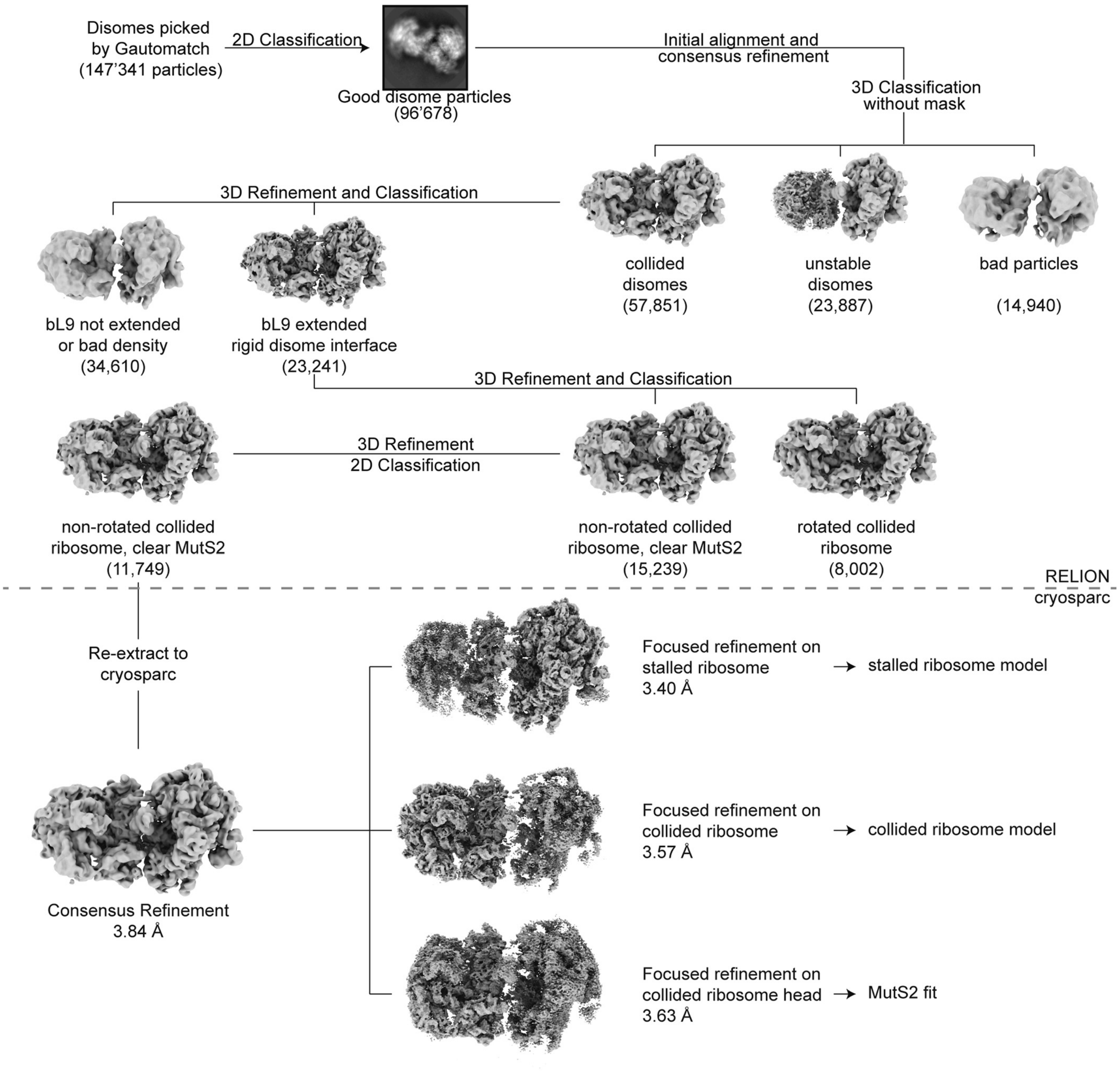

Figure EV3. Processing scheme for reconstituted MutS2-disome complex.

Shown are the principal steps of processing as well as representative reconstructions for each step. Initial processing steps and classification were performed in Relion, followed by high-resolution refinement of the resulting class of particles in CryoSPARC.

 © The Author(s)

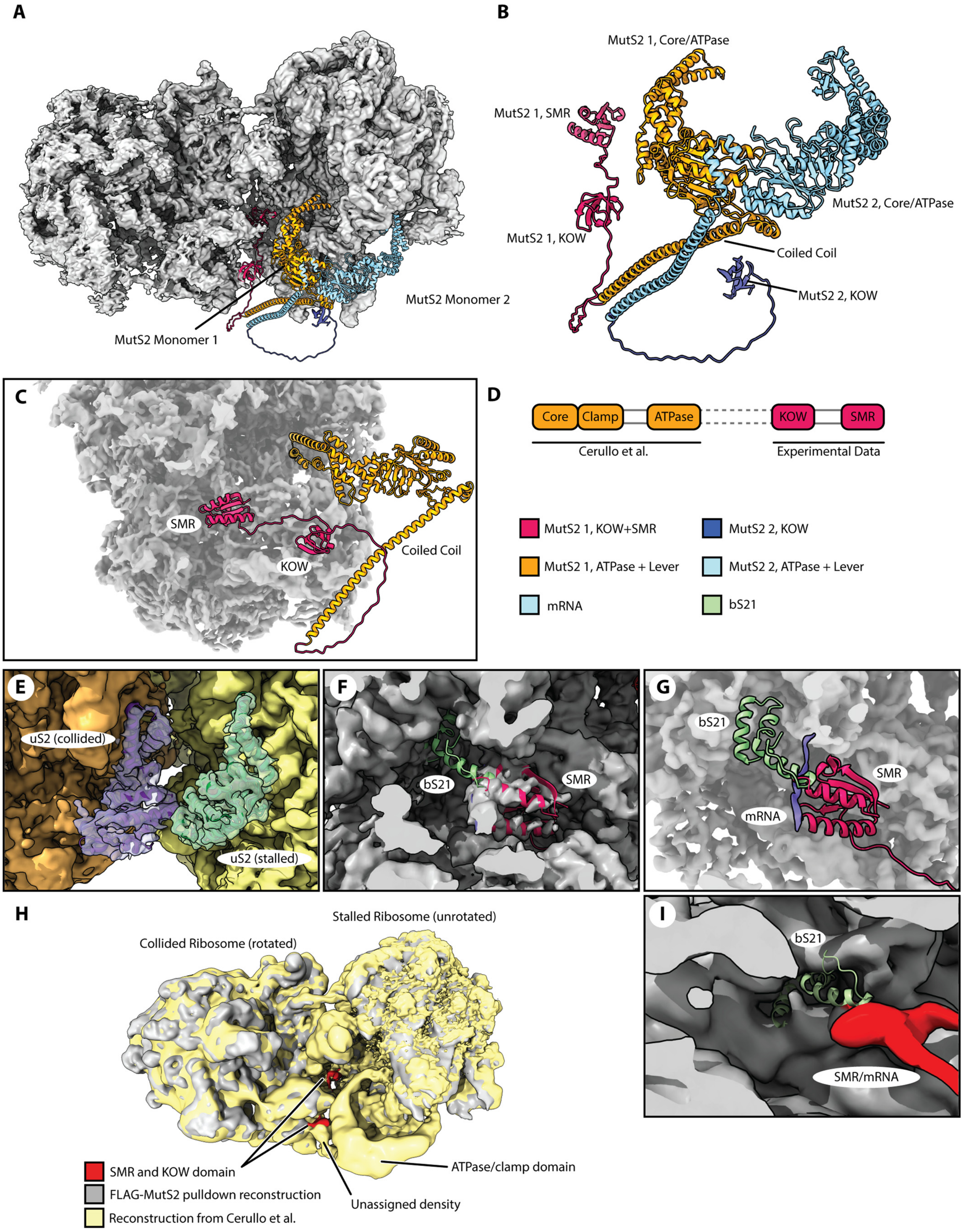
A
MutS2 Monomer 2
MutS2 Monomer 1
B
MutS2 1, Core/ATPase
MutS2 1, SMR
MutS2 2, Core/ATPase
MutS2 1, KOW
Coiled Coil
MutS2 2, KOW
C
SMR
KOW
Coiled Coil
D
Core
Clamp
ATPase
KOW
SMR
Cerullo et al.
Experimental Data
MutS2 1, KOW+SMR
MutS2 2, KOW
MutS2 1, ATPase + Lever
MutS2 2, ATPase + Lever
mRNA
bS21
E
uS2 (collided)
uS2 (stalled)
F
bS21
SMR
G
bS21
SMR
mRNA
H
Stalled Ribosome (unrotated)
Collided Ribosome (rotated)
SMR and KOW domain
FLAG-MutS2 pulldown reconstruction
Reconstruction from Cerullo et al.
ATPase/clamp domain
Unassigned density
I
bS21
SMR/mRNA

Figure EV4. MutS2 KOW and SMR domains bind the ribosome in a manner congruent with previous studies on the MutS2 Core/ATPase domains.

(**A**) Experimental cryo-EM map (gray) and model of the MutS2 dimer binding a collided disome in *B. subtilis*. The SMR and KOW domains of MutS2 monomer 1 (red) as well as the KOW domain of MutS2 monomer 2 (violet) recruit the MutS2 are visible in the cryo-EM reconstruction. The Core/ATPase domains are not visible in the reconstruction, but the structure as published by Cerullo et al (monomer 1: yellow, monomer 2: light blue) is congruent with our experimental observations. (**B**) Isolated view of the composite structure of the MutS2 dimer: The length of the flexible loop between coiled-coil and KOW domains does not allow a stringent assignment of either KOW domain to either monomer from the Cerullo et al structure, hence the assignment shown here was chosen arbitrarily. (**C**) Side view of MutS2 monomer 1 engaged with the stalled ribosome. (**D**) Schematic representation of the composite structure of MutS2 shown in (**A**, **B**). (**E**) Map-to-model fit of uS2 from both stalled and collided ribosome to a composite map of the MutS2-bound *B. subtilis* disome. (Stalled and collided ribosome maps were refined separately). uS2 is clearly present on both ribosomes in the complex. (**F**) Fit of the MutS2 monomer 1 SMR domain into the experimental density and comparison with the hypothetical location of bS21 as observed by Cerullo et al. In our experimental data, there is no evidence that bS21 is present in the MutS2-bound collided disomes. (**G**) Representation of the experimentally determined location of the MutS2 monomer 1 SMR domain and the position of bS21 as observed in Cerullo et al. bS21 would clash with the observed conformation of MutS2 SMR next to the mRNA. (**H**) Overlay of the experimental cryo-EM map of the MutS2-bound disome published by Cerullo et al and the experimental map of disomes collected from a FLAG-MutS2 pulldown in our experiments. SMR and KOW domain are highlighted in our experimental map. Both ribosomes match in their rotation states, the stalled ribosome being non-rotated in both maps and the collided ribosome rotated. MutS2 ATPase/Core/Clamp domains are visible only in the reconstruction from Cerullo et al, while the SMR domain is visible in our reconstruction. An extra density in the map of Cerullo et al that was not assigned in the original publication is visible close to the position of the KOW domain identified in our data. (**I**) Close-up view of the binding pocket for bS21 in our in vivo dataset. Only very weak partial density can be observed for bS21 compared to surrounding ribosomal proteins and the density corresponding to mRNA and the MutS2 SMR domain.

 © The Author(s)

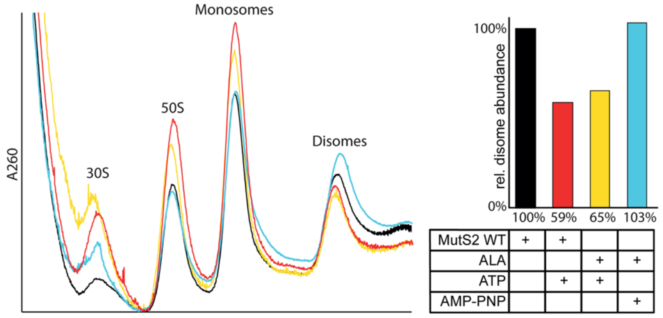

Figure EV5. The DLR motif of MutS2 SMR domain is not essential for disome splitting.

Left: UV chromatograms from sucrose gradient fractionation of disome splitting assays with MutS2 WT and MutS2 $D_{711}LR$ to $A_{711}LA$ mutant ("ALA"). Right: Relative abundance of disomes compared to total ribosomal fractions after splitting reaction, calculated from relative peak areas in the chromatograms. Purified *B. subtilis* disomes were used as input. The presence of the mutation has no effect on the efficiency of the splitting reaction either with or without hydrolysable ATP, indicating that the DLR motif of the SMR domain is not required for this process.

© The Author(s)
